# *Plag1* and *Plagl2* have overlapping and distinct functions in telencephalic development

**DOI:** 10.1242/bio.038661

**Published:** 2018-10-25

**Authors:** Lata Adnani, Rajiv Dixit, Xingyu Chen, Anjali Balakrishnan, Harshil Modi, Yacine Touahri, Cairine Logan, Carol Schuurmans

**Affiliations:** 1 Sunnybrook Research Institute, Biological Sciences, Room S1-16A, 2075 Bayview Ave, Toronto, ON, Canada M4N 3M5; 2 Department of Biochemistry and Molecular Biology, Alberta Children's Hospital Research Institute and Hotchkiss Brain Institute, University of Calgary, Calgary, Alberta T2N 4N1, Canada; 3 Department of Biochemistry, University of Toronto, Toronto, Ontario, Canada M5S 1A8; 4 Department of Cell Biology and Anatomy, University of Calgary, 3330 Hospital Drive NW, Calgary, Alberta, Canada T2N 4N1

**Keywords:** *Plag* gene family, Zinc finger transcription factors, Neocortical development, Neural progenitor proliferation, Neurogenesis, Telencephalic patterning

## Abstract

The *Plag* gene family has three members; *Plagl1/Zac1*, which is a tumor suppressor gene, and *Plag1* and *Plagl2*, which are proto-oncogenes. All three genes are known to be expressed in embryonic neural progenitors, and *Zac1* regulates proliferation, neuronal differentiation and migration in the developing neocortex. Here we examined the functions of *Plag1* and *Plagl2* in neocortical development. We first attempted, and were unable to generate, E12.5 *Plag1;Plagl2* double mutants, indicating that at least one *Plag1* or *Plagl2* gene copy is required for embryonic survival. We therefore focused on single mutants, revealing a telencephalic patterning defect in E12.5 *Plagl2* mutants and a proliferation/differentiation defect in *Plag1* mutant neocortices. Specifically, the ventral pallium, a dorsal telencephalic territory, expands into the ventral telencephalon in *Plagl2* mutants. In contrast, *Plag1* mutants develop normal regional territories, but neocortical progenitors proliferate less and instead produce more neurons. Finally, in gain-of-function studies, both *Plag1* and *Plagl2* reduce neurogenesis and increase BrdU-uptake, indicative of enhanced proliferation, but while *Plagl2* effects on proliferation are more immediate, *Plag1* effects are delayed. Taken together, we found that the *Plag* proto-oncogenes genes are essential regulators of neocortical development and although *Plag1* and *Plagl2* functions are similar, they do not entirely overlap.

This article has an associated First Person interview with the first author of the paper.

## Abstract (precis)

**Summary:** *Plag1* and *Plagl2* are proto-oncogenes that have been studied extensively in cancer. Here we provide the first report of a role for these genes in the developing central nervous system.

## INTRODUCTION

The *Pleomorphic adenoma gene* (*Plag*) family includes three genes: *Plag-like 1* (*Plagl1*; also known as *Zac1*), *Plag1* and *Plagl2*. *Plag* genes encode C_2_H_2_ Zn-finger transcription factors that are key regulators of tumorigenesis (Abdollahi, 2007; Van Dyck et al., 2007b). *Zac1* was initially identified as a gene lost on transformation (*Lot1*) in a spontaneously transformed cell line (Abdollahi et al., 1997a). Human *ZAC1* was subsequently found to be located on 6q24-25, a locus silenced in multiple carcinomas, including head and neck, ovarian, breast, kidney and pituitary tumors (Abdollahi et al., 1997b; Chappell et al., 1997; Colitti et al., 1998; Cvetkovic et al., 2004; Kamikihara et al., 2005; Koy et al., 2004; Lemeta et al., 2007; Pagotto et al., 2000; Poulin and Labelle, 2005; Theile et al., 1996; Theodoropoulou et al.; Theodoropoulou et al., 2009, 2006; Varrault et al., 1998). Consistent with its role as a tumor suppressor gene, *Zac1* promotes cell cycle exit and apoptosis *in vitro* in various cell lines (Bilanges et al., 2001; Pagotto et al., 1999; Spengler et al., 1997; Varrault et al., 1998) as well as *in vivo* in the developing nervous system (Adnani et al., 2015; Ma et al., 2007b; Rraklli et al., 2016).

In contrast to *Zac1*, *Plag1* and *Plagl2* function as proto-oncogenes (Hensen et al., 2002). *Plag1* has been shown to be amplified in pleomorphic adenomas of the salivary gland (Asp et al., 2006; Astrom et al., 1999; Debiec-Rychter et al., 2001; Enlund et al., 2002; Kandasamy et al., 2007; Kas et al., 1997; Voz et al., 1998), lipoblastomas (Astrom et al., 2000; Gisselsson et al., 2001; Hibbard et al., 2000; Morerio et al., 2005; Röpke et al., 2007), hepatoblastomas (Zatkova et al., 2004) and some leukemias (Landrette et al., 2005; Pallasch et al., 2009). The misexpression of *Plag1* in these cancers is due to chromosomal translocations that place *Plag1* under the control of regulatory elements for ubiquitously expressed genes, such as *Elongation factor SII gene* (*Tcea1*) (Colitti et al., 1998), *Ctnnb1* (*β-catenin*) (Valente et al., 2005) and *Leukemia inhibitory factor receptor* (*Lifr*) (Cvetkovic et al., 2004). *Plagl2* is similarly amplified in a number of cancers, including glioblastomas (Zheng et al., 2010) and acute myeloid leukemia (Landrette et al., 2005). Consistent with their roles as oncogenes, *Plag1* and *Plagl2* promote proliferation, anchorage-independent growth, loss of contact inhibition and tumor formation in mice (Declercq et al., 2003, 2005; Hensen et al., 2002; Landrette et al., 2005; Van Dyck et al., 2008; Zhao et al., 2006; Zheng et al., 2010). However, *Plagl2* is not oncogenic in all contexts as it is pro-apoptotic in response to hypoxia and other cellular stresses (Furukawa et al., 2001; Guo et al., 2007; Juma et al., 2016; Mizutani et al., 2002; Yang et al., 2009).

All three members of the *Plag* gene family encode zinc finger transcription factors that share homology chiefly in their amino terminal zinc (Zn) finger domains, whereas the carboxyl terminal regions of the three proteins are quite diverse (Kas et al., 1998). Several transcriptional targets of the Plag family transcription factors have been identified. For example, *Plag1* and *Plagl2* both regulate the expression of *Insulin-like Growth Factor 2* (*Igf2*), which accounts at least in part for their abilities to stimulate cell proliferation (Ciani et al., 2003; Declercq et al., 2008; Varrault et al., 1998; Voz et al., 2000). In addition, *Plag1/Plagl2* promote tumorigenesis by initiating the transcription of several Wnt pathway genes. For instance, *Plagl2* has been shown to regulate expression of *Wnt6*, *Fzd2* and *Fzd9* to maintain cells in a proliferative state (Zheng et al., 2010). Likewise, *Plag1* misexpression in pleomorphic adenomas results in an upregulation of canonical Wnt signaling (Declercq et al., 2008; Zhao et al., 2006). Finally, *Plag1* was also found to regulate several cell division and cell cycle-related genes, such as *Cyclin D3* and *Cyclin D1*, as well as apoptosis-related genes, such as *Caspase-8* (Voz et al., 2004).

Despite extensive knowledge of *Plag* gene function in cancer, their roles during normal development have only recently been examined. Z*ac1*, *Plag1* and *Plagl2* all function to regulate embryonic growth (Hensen et al., 2004; Van Dyck et al., 2007a; Varrault et al., 2006). *Zac1* also controls development of keratinocytes (Basyuk et al., 2005), heart (Czubryt et al., 2010; Yuasa et al., 2010) and pancreatic islets (Anderson et al., 2009), while *Plagl2* functions to control the development of enterocytes (Van Dyck et al., 2007a). All three *Plag* genes are expressed in several lineages in the developing embryo as well as in some adult tissues, each with unique expression domains that overlap in certain lineages/tissues (Alam et al., 2005; Hensen et al., 2004; Rodríguez-Henche et al., 2002; Van Dyck et al., 2007a). For example, *Zac1* is expressed in a regionalized fashion in neural progenitor cells in the developing central (CNS) and peripheral (PNS) nervous systems, whereas *Plag1* and *Plagl2* are more uniformly expressed in CNS and PNS neural progenitors (Abdollahi, 2007; Alam et al., 2005; Astrom et al., 1999; Poulin and Labelle, 2005). Interestingly, all three *Plag* genes are co-expressed at higher levels in neural progenitors than in post-mitotic neurons (Alam et al., 2005; Rodríguez-Henche et al., 2002).

*Plag1* null mice (*Plag1*^KI/KI^), although viable, are growth retarded and have reduced fertility (Hensen et al., 2004). However, despite their growth defects and the known ability of *Plag1* to regulate expression of the *Igf2* growth factor (Voz et al., 2000), *Igf2* expression levels were found to be unperturbed in *Plag1* null mice (Hensen et al., 2004). Thus, the underlying molecular mechanisms that lead to growth perturbation in *Plag1* null embryos remain unknown. Likewise, *Plagl2* mutant neonates also weigh less relative to their littermates at birth (Van Dyck et al., 2007a). However, unlike *Plag1* mutants, *Plagl2* mutant pups display postnatal lethality, dying shortly after birth due to starvation and nutrient malabsorption (Van Dyck et al., 2007a). In the neonatal *Plagl2* mutant liver, the starvation response factor *asparagine synthetase* is expressed at high levels (Van Dyck et al., 2007a), whereas *Igf1* levels are low, indicative of a loss of nutrients.

In the CNS, multiple developmental roles for *Zac1* have been deciphered, including in the retina, cerebellum and neocortex (Adnani et al., 2015; Chung et al., 2011; Ma et al., 2007a,b; Rraklli et al., 2016). However, to date, neither *Plag1* nor *Plagl2* have any known functions in the developing CNS. Here, given their overlapping expression with *Zac1*, we asked whether *Plag1* and *Plagl2*, also function during neocortical development, revealing novel and specific roles for these genes in both telencephalic patterning and in regulating neocortical progenitor cell proliferation and neurogenesis.

## RESULTS

### *Plag1* and *Plagl2* do not cross-regulate each other at the level of transcription

*Plag1* and *Plagl2* have similar amino acid sequences, sharing 79% and 35% identity in their N- and C-termini, respectively (Juma et al., 2016). They also share several transcriptional targets, including the growth factor *Igf2* (Abdollahi, 2007). In addition, *Plag1* and *Plagl2* have both been characterized as growth regulators and proto-oncogenes (Abdollahi, 2007; Juma et al., 2016; Landrette et al., 2005). Here we set out to determine whether they also have overlapping and possibly redundant roles in the developing telencephalon.

**Fig. 1.**
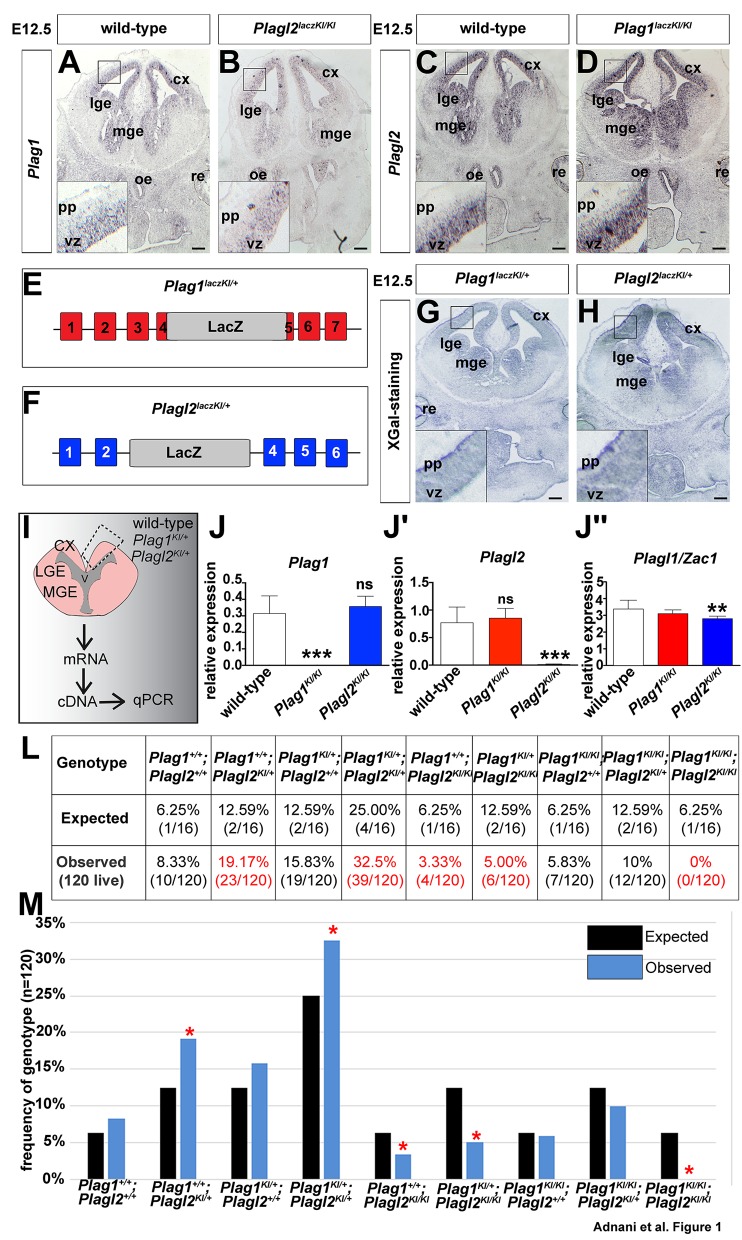
***Plag1* and *Plagl2* have similar patterns of telencephalic gene expression and function redundantly to regulate embryonic development.** (A–D) Expression of *Plag1* (A,B) and *Plagl2* (C,D) in E12.5 wild-type (A,C), *Plagl2^lacZ^*^KI/KI^ (B) and *Plag1^lacZ^*^KI/KI^ (D) whole heads. (E,F) Schematic representations of genetic mutations in *Plag1^lacZ^*^KI/KI^ (E) and *Plagl2^lacZ^*^KI/KI^ (F) mice (G,H) X-gal staining of E12.5 *Plagl^lacZ^*^KI/+^ (G) and *Plagl2^lacZ^*^KI/+^ (H) brains. (I–J″) Schematic representation of RT-qPCR experiment (I). Analysis of *Plag1* (J), *Plagl2* (J′) and *Plagl1*/*Zac1* (J″) transcript levels in E12.5 wild-type, *Plag1*^KI/KI^ and *Plagl2*^KI/KI^ cortices. ns, not significant; *P<0.05, **P<0.01, and ***P<0.005. (L,M) Punette square analysis of the ratios of genotypes acquired from *Plag1*^KI/+^;*Plagl2*^KI/+^ heterozygous intercrosses (L). Graphical representation of the expected (black bars) and observed (blue bars) numbers of embryos with each genotype (M). cx, neocortex; lge, lateral ganglionic eminence; mge, medial ganglionic eminence; oe, olfactory epithelium; pp, preplate; re, retina; vz, ventricular zone. Scale bars: 250 μm.

To better understand how *Plag1* and *Plagl2* function in the embryonic telencephalon, we first examined their expression profiles at embryonic day (E) 12.5, when the first neurons have begun to differentiate in both dorsal and ventral domains (Adnani et al., 2018). As previously reported (Alam et al., 2005), *Plag1* (Fig. 1A) and *Plagl2* (Fig. 1C) were expressed in E12.5 telencephalic progenitors throughout the dorsal and ventral ventricular zones (VZ) in a highly similar fashion, albeit with apparently higher *Plagl2* transcript levels. We also monitored the expression of these genes by taking advantage of the knockin of *lacZ* into the *Plag1* [*Plag1^lacZ^*^KI/+^; Fig. 1E; Hensen et al. (2004)] and *Plagl2* [*Plagl2^lacZ^*^KI/+^; Fig. 1F; Van Dyck et al. (2007a)] loci. X-gal staining of coronal sections of E12.5 *Plag1^lacZ^*^KI/+^ (hereafter *Plag1*^KI/+^; Fig. 1G) and *Plagl2^lacZ^*^KI/+^ (hereafter *Plagl2*^KI/+^; Fig. 1H) heterozygous brains revealed that *lacZ* had a similar distribution throughout the telencephalic VZ in both genotypes, again with apparently higher levels of *lacZ* expression in *Plagl2*^KI/+^ cortices. *Plag1* and *Plagl2* are thus expressed similarly in the early embryonic telencephalic VZ, an expression profile that we previously demonstrated persists into the late embryonic period (Alam et al., 2005).

In several instances, highly related genes in the same family are not only expressed in the same CNS domains, but also display cross-regulatory interactions. For example, the highly similar proneural genes *Neurog1* and *Neurog2* are largely co-expressed in the early embryonic dorsal telencephalon (Britz et al., 2006; Han et al., 2018), and *Neurog2* is required to initiate *Neurog1* expression in the dorsomedial cortex (Fode et al., 2000). To determine whether there are similar cross-regulatory interactions between *Plag1* and *Plagl2* in the developing telencephalon, we asked whether mutation of one gene can alter expression of the other gene. By RNA *in situ* hybridization, we observed a similar distribution of *Plag1* transcripts in the telencephalic VZ of E12.5 *Plagl2*^KI/KI^ homozygous mutants as seen in wild-type brains (Fig. 1A,B). The converse was also true, as *Plagl2* transcripts were maintained in the telencephalic VZ of E12.5 *Plag1*^KI/KI^ homozygous mutants in a similar pattern as in wild-type brains (Fig. 1C,D).

To validate that there were no differences in *Plag1* and *Plagl2* transcript levels in homozygous mutants for the other *Plag* gene, we micro-dissected out telencephalic tissue at E12.5 and performed RT-qPCR (Fig. 1I). As expected, *Plag1* transcripts were not detectable in *Plag1*^KI/KI^ cortices, whereas *Plag1* transcript levels were at the same relative level in wild-type controls and in E12.5 *Plagl2*^KI/KI^ homozygous mutant cortices (Fig. 1J). Similarly, *Plagl2* transcripts were not detectable in cortical tissue from *Plagl2*^KI/KI^ homozygous mutants, whereas *Plagl2* transcripts were expressed at wild-type levels in E12.5 *Plag1*^KI/KI^ cortices (Fig. 1J′). To complete this data set, we also analyzed expression levels of the third member of this gene family, *Plagl1* (also known as *Zac1*), which differs in that it is a tumor suppressor gene (Abdollahi, 2007). *Zac1* is expressed at high levels in neocortical progenitors and plays a role in regulating neuronal morphology and migration (Adnani et al., 2015). While no differences in *Zac1* transcript levels were observed in *Plag1*^KI/KI^ cortices, there was a small but significant reduction in *Zac1* expression in *Plagl2*^KI/KI^ dorsal telencephalic tissue (Fig. 1J″, *P*<0.01, *n*=3). Finally, a comparison of relative transcript levels (normalized to the same housekeeping genes) revealed that *Zac1* is expressed at the highest levels in cortical cells, followed by *Plagl2* and *Plag1*.

Thus, all three *Plag* genes are expressed in cortical progenitors, and while there is no evidence of cross-regulatory transcriptional interactions between *Plag1* and *Plagl2*, *Zac1* levels are reduced in *Plagl2* mutants, at least at E12.5, which is the stage we focused on for the remainder of this study.

### *Plag1* and *Plagl2* act redundantly to control embryonic survival

Previous reports have suggested that *Plag1*^KI/KI^ null mice are viable after birth, but are growth retarded and have reduced fertility (Hensen et al., 2004). Likewise, *Plagl2*^KI/KI^ neonates weigh less relative to their littermates at birth (Van Dyck et al., 2007a). However, unlike *Plag1*^KI/KI^ mutants, *Plagl2^KI/KI^* pups display postnatal lethality, dying shortly after birth due to starvation and nutrient malabsorption (Van Dyck et al., 2007a). To determine whether *Plag1* and *Plagl2* function redundantly or have distinct functions in the embryonic telencephalon, we set out to generate double mutants by setting up double heterozygous intercrosses between *Plag1*^KI/+^;*Plagl2*^KI/+^ male and female mice. We collected seventeen litters at E12.5 for a total of 120 live embryos and compared the acquired genotypes to the expected genotypes using a Mendelian Punette square diagram for a dihybrid cross (Fig. 1L). If there was no embryonic lethality, we expected Mendelian ratios for each possible genotype after double heterozygous intercrosses. Of the 120 embryos genotyped, significantly under-represented genotypes included *Plag1^+/+^;Plagl2^KI/KI^*, *Plag1^KI/+^;Plagl2^KI/KI^* and *Plag1^KI/KI^;Plagl2^KI/KI^* (Fig. 1M).

As we did not collect any double mutant embryos, our data suggests that *Plag1* and *Plagl2* function redundantly to control embryonic survival. Moreover, the *Plagl2^KI/KI^* genotype has an early embryonic lethal phenotype. This finding was somewhat surprising given that *Plagl2^KI/KI^* mutant embryos were previously reported to survive postnatally (Van Dyck et al., 2007a). Differences between the two studies are likely related to our use of a different genetic background (i.e. CD1) compared to 129/SvJ background used by Van Dyck et al. (2007a).

### *Plagl2* is required to set the positioning of ventral gene expression at the pallial-subpallial border

Neuronal fate specification is directly linked to dorsoventral regional identity in the telencephalon, with progenitors in the dorsal telencephalon giving rise to glutamatergic excitatory projection neurons, while ventral progenitors give rise to GABAergic inhibitory interneurons (Schuurmans and Guillemot, 2002). Given that *Plag1* and *Plagl2* were expressed in both dorsal and ventral telencephalic progenitors, we first asked whether the *Plag* genes acted upstream of regional patterning genes. Genes involved in the initial patterning of telencephalic domains are expressed in a regionalized manner and are enriched in, or restricted to, precise dorsal or ventral telencephalic progenitor domains, displaying sharp dorsoventral boundaries (Hoch et al., 2009). Mutation of several patterning genes can disrupt the positioning of borders between dorsal and ventral domains, ultimately affecting the generation of the brain territories derived from these regionalized progenitors. For instance, mutation of the homeobox gene *Gsx2* results in an expansion of dorsal telencephalic (pallial) territories and a corresponding reduction in ventral telencephalic (subpallial) territories, whereas *Pax6* mutants have the opposite phenotype (Yun et al., 2001).

**Fig. 2.**
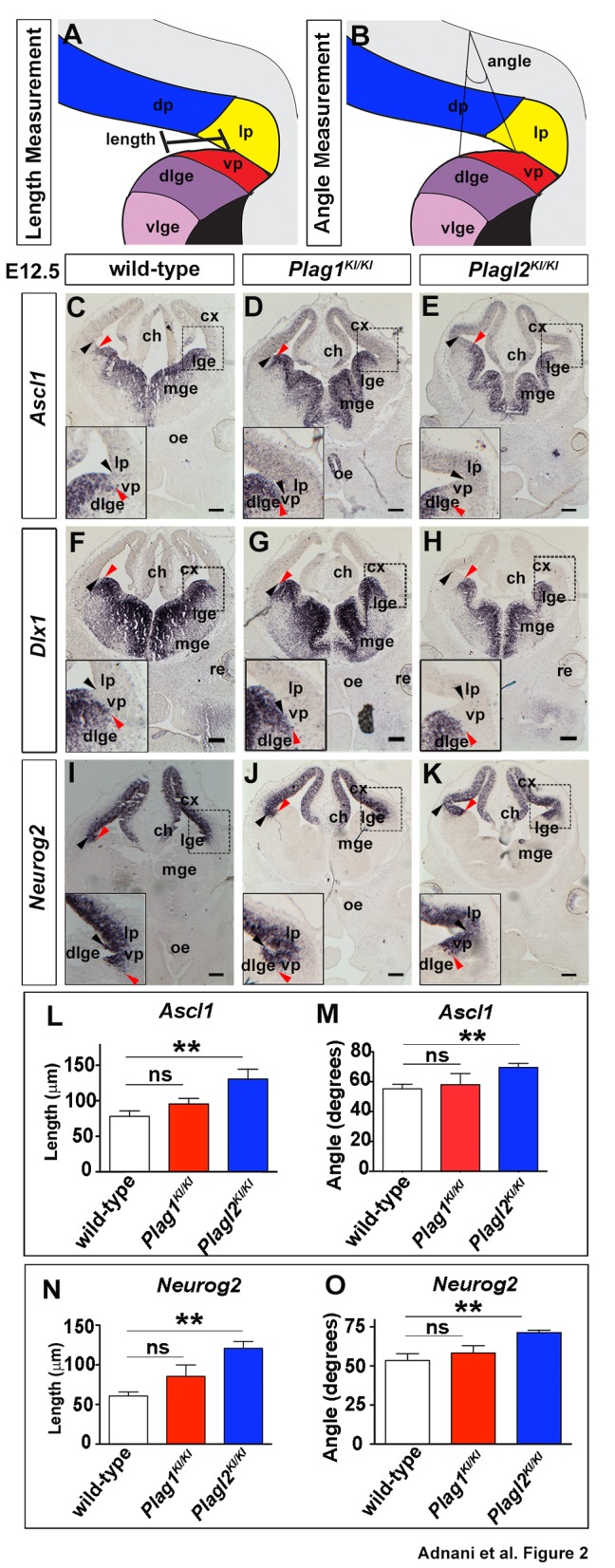
***Plag1* and *Plagl2* are required to pattern the embryonic telencephalon.** (A,B) Schematic representations of length (A) and angle (B) measurements of the ventral pallium, extending from the corticostriatal angle to the gene expression border. (C–K) Expression of *Ascl1* (C–E), *Dlx1* (F–H) and *Neurog2* (I–K) in E12.5 wild-type (C,F,I), *Plag1*^KI/KI^ (D,G,J) and *Plagl2*^KI/KI^ (E,H,K) brains. Black arrowheads mark the corticostriatal angle and red arrowheads mark the ventral pallial gene expression limit. (L–O) Quantification of the length (L,N) and angle (M,O) of the ventral pallium based on the expression of *Ascl1* (L,M), and *Neurog2* (N,O). Error bars are s.e.m. ns, not significant; *P<0.05, **P<0.01, and ***P<0.005. ch, cortical hem; cx, neocortex; dlge, dorsal lateral ganglionic eminence; dp, dorsal pallium; lge, lateral ganglionic eminence; lp, lateral pallium; mge, medial ganglionic eminence; mp, medial pallium; oe, olfactory epithelium; re, retina; vlge, ventral lateral ganglionic eminence; vp, ventral pallium. Scale bars: 250 μm.

To assess the roles of the *Plag* genes in dorsoventral patterning, we focused on *Plag1* and *Plagl2* single mutants as we were not able to generate E12.5 *Plag1;Plagl2* double mutants. In these embryos, we compared the position of the dorsoventral border between high and low gene expression in the telencephalon (red arrowheads; Fig. 2C–K) to a morphological landmark, the corticostriatal angle (black arrowheads; Fig. 2C–K). To provide quantitative measurements of regional differences, we measured the length (Fig. 2A) and angle (Fig. 2B) between the corticostriatal border (morphological landmark) and the pallial-subpallial boundary (PSPB, gene expression landmark), using a fixed lever length for angle measurements.

We first examined the expression of *Ascl1*, a proneural gene encoding a basic-helix-loop-helix (bHLH) transcription factor that is required for ventral telencephalic development (Casarosa et al., 1999). At E12.5, *Ascl1* was expressed at high levels in the ventral telencephalic VZ, including in both the lateral (LGE) and medial (MGE) ganglionic eminences (Fig. 2C), as previously reported (Casarosa et al., 1999). *Ascl1* transcripts were also enriched in the cortical hem, while lower levels of *Ascl1* transcripts were detected in the dorsal telencephalic VZ (Fig. 2C). The dorsal most limit of the high *Ascl1*-expression domain marked the PSPB; immediately ventral to the PSPB was an *Ascl1*-high subpallial territory known as the dorsal LGE (dLGE) and immediately dorsal to the PSPB was an *Ascl1*-low pallial territory known as the ventral pallium (Yun et al., 2001) (Fig. 2C). A similar pattern of expression was observed in E12.5 *Plag1*^KI/KI^ mutant brains (Fig. 2D), while the high *Ascl1* expression domain appeared to shift ventrally in E12.5 *Plagl2*^KI/KI^ cortices (Fig. 2E). Indeed, measurement of the length (*n*=3; *P*<0.01; Fig. 2L) and angle (*n*=3; *P*<0.01; Fig. 2M) between the corticostriatal angle and the limit of the high *Ascl1* expression domain confirmed that these values were greater in the E12.5 *Plagl2* telencephalon compared to wild-type brains.

To provide further validation for this finding, we also examined the expression of *Dlx1*, a homeodomain transcription factor that acts with the related gene *Dlx2* to establish a ventral telencephalic identity, with the absence of these genes, resulting in the loss of most if not all GABAergic interneurons (Anderson et al., 1997). In E12.5 wild-type brains, *Dlx1* was expressed at high levels throughout the VZ of the LGE and MGE, with its dorsal limit in the dLGE, and no expression in the ventral pallium (Fig. 2F). A similar pattern of expression was observed in the E12.5 *Plag1*^KI/KI^ telencephalon (Fig. 2G), whereas in E12.5 *Plagl2*^KI/KI^ brains (Fig. 2H), the dorsal limit of the *Dlx1* expression domain was positioned more ventrally.

Taken together, these data suggest that *Plagl2* is required to maintain the position of the PSPB in the E12.5 telencephalon, a contention that we investigated further with additional marker analysis.

### *Plagl2* is required to set the positioning of dorsal gene expression at the pallial-subpallial border

We next examined the expression of dorsally-restricted genes in E12.5 *Plag1* and *Plagl2* mutants. The proneural gene *Neurog2*, which also encodes a bHLH transcription factor, is required to specify a dorsal telencephalic identity (Fode et al., 2000). In the E12.5 wild-type telencephalon, *Neurog2* was exclusively expressed in the dorsal telencephalic VZ, with the ventral border of expression extending into the ventral pallium and ending at the PSPB (Fig. 2I). A similar pattern of *Neurog2* expression was observed in the E12.5 *Plag1*^KI/KI^ (Fig. 2J) telencephalon, while the limit of *Neurog2* expression extended ventrally in *Plagl2*^KI/KI^ brains (Fig. 2K). We validated these observations by performing measurements of the length (*n*=3; *P*<0.01; Fig. 2N) and angle (*n*=3; *P*<0.01; Fig. 2O) between the corticostriatal angle and the limit of high *Neurog2* expression.

**Fig. 3.**
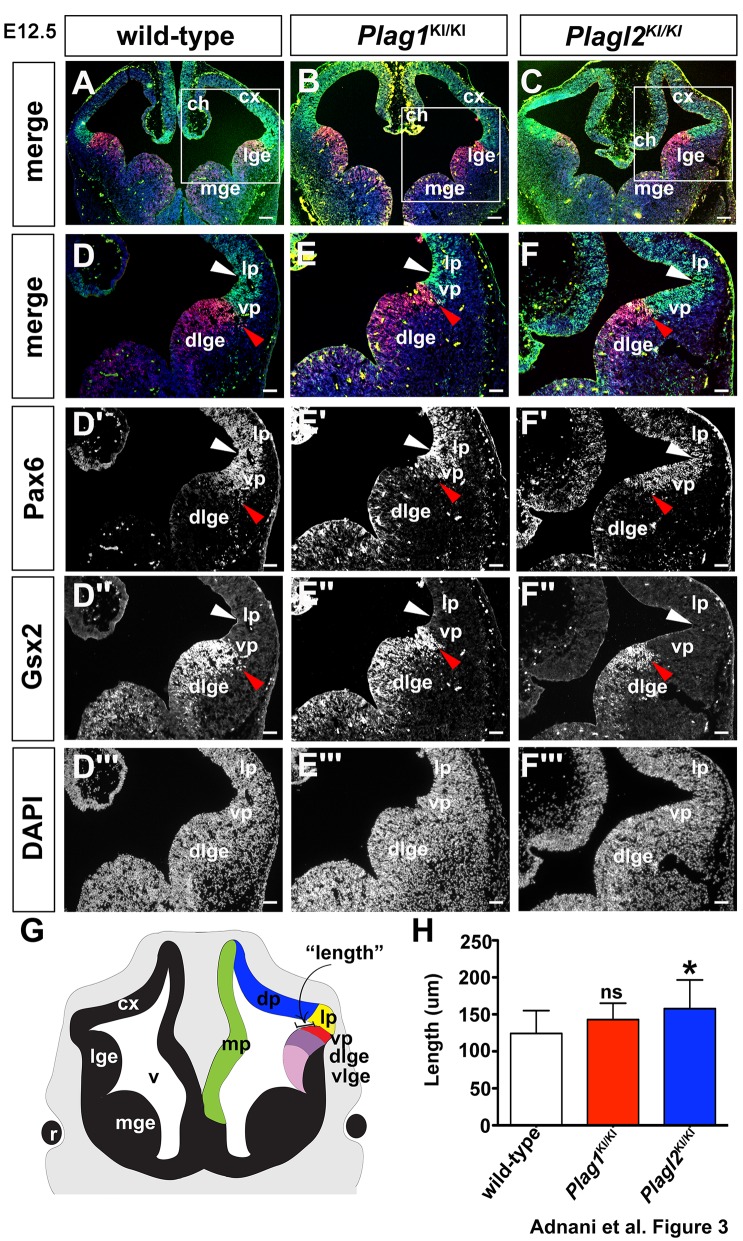
***Plagl2* is required to maintain the pallial-subpallial boundary in the developing telencephalon.** (A–F) Expression of Pax6 (green), Gsh2 (red) and DAPI (blue) in E12.5 wild-type (A,D–D‴), *Plag1*^KI/KI^ (B,E–E‴) and *Plagl2*^KI/KI^ (C,F–F‴) brains. (D′–F‴) Higher magnification images of Pax6 (D′–F′), Gsh2 (D″–F″) and DAPI (D‴–F‴) in E12.5 wild-type (D′–D‴), *Plag1*^KI/KI^ (E′–E‴) and *Plagl2*^KI/KI^ (F′–F‴) brains. White arrowheads mark the corticostriatal angle. Red arrowheads mark the ventral pallium-dlge boundary. (G) Schematic illustration of the length measurement of the ventral pallium. (H) Quantification of the length of the ventral pallium. Error bars are s.e.m. ns, not significant; *P<0.05, **P<0.01, and ***P<0.005. ch, cortical hem; cx, neocortex; dlge, dorsal lateral ganglionic eminence; dp, dorsal pallium; lge, lateral ganglionic eminence; lp, lateral pallium; mge, medial ganglionic eminence; mp, medial pallium; oe, olfactory epithelium; re, retina; vlge, ventral lateral ganglionic eminence; vp, ventral pallium. Scale bars: 250 μm.

To further examine the position of the PSPB, we performed co-staining of a dorsal pallial marker, the homeodomain transcription factor Pax6, and a ventral marker, the homeodomain transcription factor Gsx2 (Fig. 3). While Pax6 expression extends into the Gsx2^+^ dLGE territory at E10.5, resulting in the co-expression of these two transcription factors in the dLGE (Yun et al., 2001), we found that at E12.5, Pax6 and Gsx2 expression domains directly abutted one another at the PSPB; Pax6 was expressed at high levels throughout the pallium, including in the ventral pallium, whereas Gsx2 was expressed exclusively in the LGE and MGE, including in the dLGE (Fig. 3A,D–D‴). A similar pattern of co-expression was observed in E12.5 *Plag1*^KI/KI^ telencephalons; high Pax6 expression in the pallium and Gsx2 expression restricted to the LGE/MGE, with little to no overlap at the PSPB (Fig. 3B,E–E‴). In contrast, in the *Plagl2*^KI/KI^ telencephalon Pax6 expression extended further ventrally, while the Gsx2 expression limit was restricted to a more ventral position, but there was still limited overlap between these markers (Fig. 3C,F–F‴). To confirm these apparent differences in expression, we quantitated the distance between the corticostriatal angle and the border between the Gsx2 and Pax6 expression domains (presumptive PSPB; Fig. 3G), revealing that this distance was indeed larger in the *Plagl2*^KI/KI^ telencephalon (*n*=3; *P*<0.05; Fig. 3H).

### Expansion of the ventral pallium in *Plagl2* mutants

**Fig. 4.**
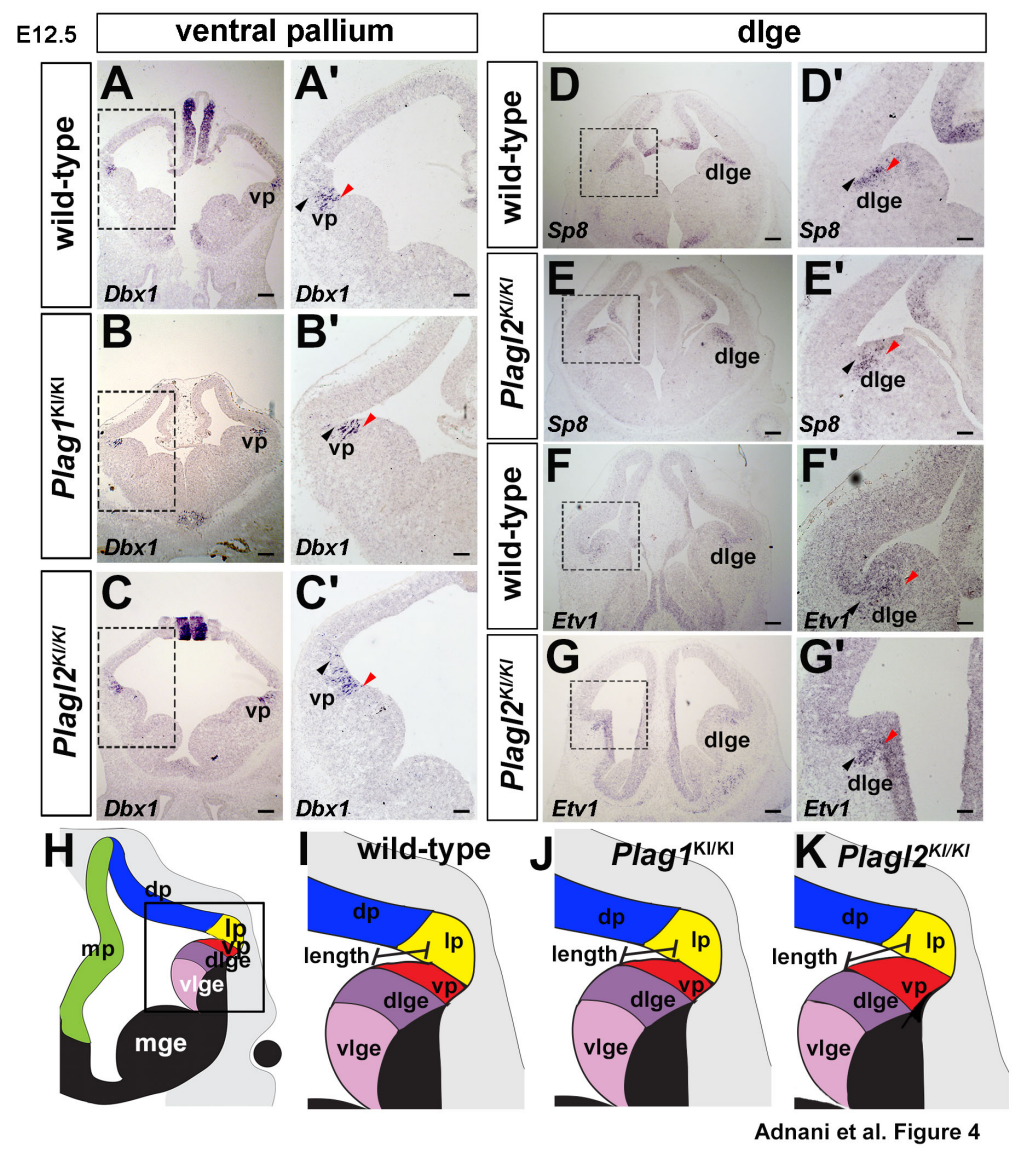
***Plagl2* is required to regulate the ventral pallium boundary in the developing telencephalon.** (A–C′) Expression of the ventral pallial gene, *Dbx1*, in E12.5 wild-type (A,A′), *Plag1*^KI/KI^ (B,B′) and *Plagl2*^KI/KI^ (C,C′) telencephalons. (D–G′) Expressions of the dlge markers, *Sp8* (D–E′) and *Etv1* (F–G′) in E12.5 wild-type (D,D′,F,F′) and *Plagl2*^KI/KI^ (E,E′,G,G′) telencephalons. Black arrowheads mark the corticostriatal angle. Red arrowheads mark the dorsal limit of gene expression. (H–K) Schematic illustration of the pallial-subpallial boundary between the ventral pallium and dlge in E12.5 wild-type (H,I), *Plag1*^KI/KI^ (J) and *Plagl2*^KI/KI^ (K), showing positioning defects in the *Plagl2*^KI/KI^ brains. dlge, dorsal lateral ganglionic eminence; vp, ventral pallium. Scale bars: 250 μm.

The shift in the positioning of the PSPB could mean that there was an expansion in the size of the ventral pallium, and possibly a corresponding reduction in the size of the dLGE, as observed in *Gsx2* mutants (Yun et al., 2001). To test this possibility more fully, we examined the expression of markers that specifically label these two regional territories, using *Dbx1* to label the ventral pallium (Bielle et al., 2005), and *Etv1/ER81* (Yun et al., 2001) and *Sp8* (Waclaw et al., 2006) to mark the dLGE. In E12.5 wild-type cortices, *Dbx1* labeled a small stripe of cells ventral to the corticostriatal angle, which is the ventral pallium (Fig. 4A,A′). The *Dbx1*^+^ ventral pallial territory was of a similar size and position in E12.5 *Plag1*^KI/KI^ brains (Fig. 4B,B′). In contrast, in E12.5 *Plagl2*^KI/KI^ brains, *Dbx1* expression initiated in the same position just ventral to the corticostriatal angle, but it extended further ventrally, consistent with the idea that the ventral pallium is expanded in size in *Plagl2* mutants (Fig. 4C,C′). Finally, to determine whether there was a compensatory decrease in the size of the dLGE, we examined the expression of *Sp8*, which marked a thin, similarly sized ventral stripe of cells in both E12.5 wild-type (Fig. 4D,D′) and *Plagl2*^KI/KI^ (Fig. 4E,E′) brains. In addition, no noticeable differences were observed in the *Etv1*^+^ dLGE domain size in E12.5 wild-type (Fig. 4F,F′) and *Plagl2*^KI/KI^ (Fig. 4G,G′) brains.

Taken together these data support the idea that only the ventral pallium is expanded in *Plagl2* mutants, without a corresponding change in dLGE size (Fig. 4H–K).

### Altered positioning of the pallial-subpallial border extends into intermediate neuronal progenitors but does not affect the number of early-born piriform cortex neurons

**Fig. 5.**
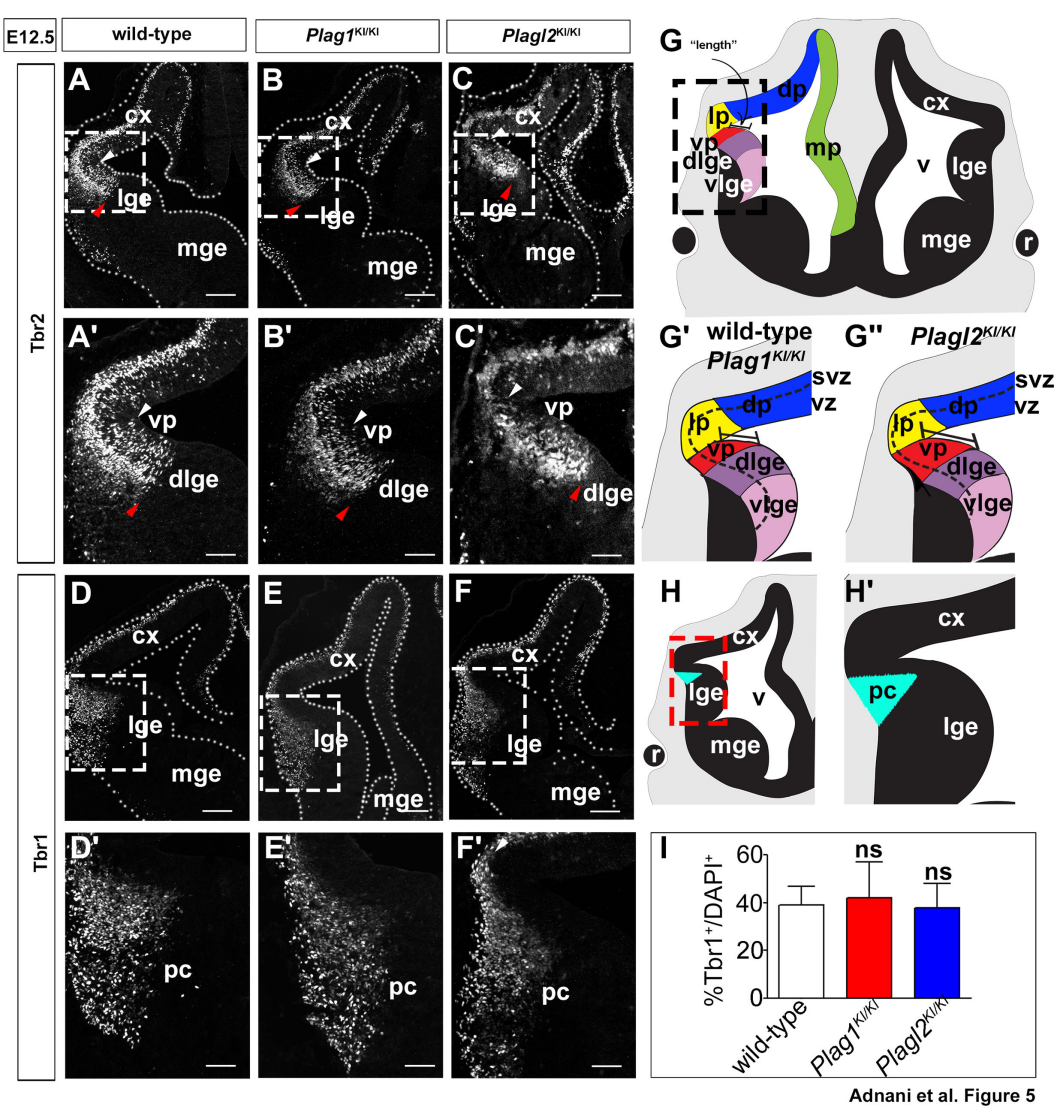
**Tbr2 expression in intermediate neuronal progenitors in the ventral pallium shifts ventrally in *Plagl2* mutants.** (A–C′) Tbr2 expression in E12.5 wild-type (A,A′), *Plag1*^KI/KI^ (B,B′) and *Plagl2*^KI/KI^ (C,C′) telencephalons. (D–F′) Tbr1 expression in E12.5 wild-type (D,D′), *Plag1*^KI/KI^ (E,E′) and *Plagl2*^KI/KI^ (F,F′) telencephalons. White arrowheads mark the corticostriatal angle. Red arrowheads mark the ventral limit of high dorsal gene expression. (G–G″) Schematic illustration of ventral pallial domain in wild-type (G,Gʹ) and *Plagl2*^KI/KI^ (G″) telencephalons. (H–Hʹ) Schematic illustration of the Tbr1 piriform cortex (blue) that was used for quantification. (I) Quantification of the Tbr1^+^/DAPI^+^ cells in the presumptive piriform cortex. Error bars are s.e.m. ns, not significant. cx, neocortex; dlge, dorsal lateral ganglionic eminence; lge, lateral ganglionic eminence; mge, medial ganglionic eminence; pc, piriform cortex; vp, ventral pallium. Scale bars: 250 μm.

Until now, our marker analyses were restricted to VZ progenitors, which are primarily radial glial cells (RGCs) that serve as progenitors for glutamatergic neurons [reviewed in Wilkinson et al. (2013)]. We next asked whether the expansion of the pallial domains in *Plagl2* mutants translated into differences in secondary pallial progenitors, the Tbr2^+^ intermediate neuronal progenitors (INPs) that are derived from Pax6^+^ RGCs (Englund et al., 2005). Although Tbr2 is expressed in the subventricular zone (SVZ), it has the same dorsoventral regional borders as *Neurog2* and Pax6, terminating at the ventral limit of the ventral pallium with no expression observed in the dLGE in E12.5 wild-type telencephalons (Fig. 5A,A′). A similar pattern of Tbr2 expression was observed in E12.5 *Plag1*^KI/KI^ brains (Fig. 5B,B′), whereas in E12.5 *Plagl2*^KI/KI^ telencephalons, Tbr2 expression extended further ventrally (Fig. 5C,C′). These data thus suggest that the increased size of the ventral pallial territory extends from VZ progenitors and into SVZ progenitors in E12.5 *Plagl2*^KI/KI^ telencephalons (Fig. 5G–G″).

Finally, we asked whether there was a corresponding shift in the expression domain of Tbr1, which marks postmitotic projection neurons that are derived from pallial territories, including the ventral pallium, which gives rise to Cajal-Retzius neurons that cover the pallial surface, and to neurons that populate the presumptive piriform cortex (Dixit et al., 2014; Yun et al., 2001). We first focused on the Tbr1^+^ mantle zone in the presumptive piriform cortex, which extended in a wedge shape from the pial surface towards the limit of the SVZ at the corticostriatal angle in E12.5 wild-type embryos (Fig. 5D,D′,H,H′). Similar patterns of Tbr1 expression were observed in the mantle of the piriform cortex in E12.5 *Plag1*^KI/KI^ (Fig. 5E,E′) and *Plagl2*^KI/KI^ (Fig. 5F,F′) brains, with no reduction in the total number of Tbr1^+^ cells (*n*=3; *P*>0.05 for all comparisons; Fig. 5I).

Taken together, these data suggest that *Plagl2* is required to maintain the position of the border between the ventral pallium and dLGE at the primary VZ progenitor and secondary SVZ progenitor stage. While there was no obvious reduction in the size of the Tbr1^+^ neuronal pool in the mantle of the presumptive piriform cortex, the complex migration patterns of ventral pallium-derived neurons, which include Cajal-Retzius neurons that migrate tangentially to cover the pallial surface (Bielle et al., 2005), makes it difficult to specifically assess neuronal output from the ventral pallium.

### *Plag1* is required to regulate the differentiation and proliferation of early embryonic neocortical progenitors

**Fig. 6.**
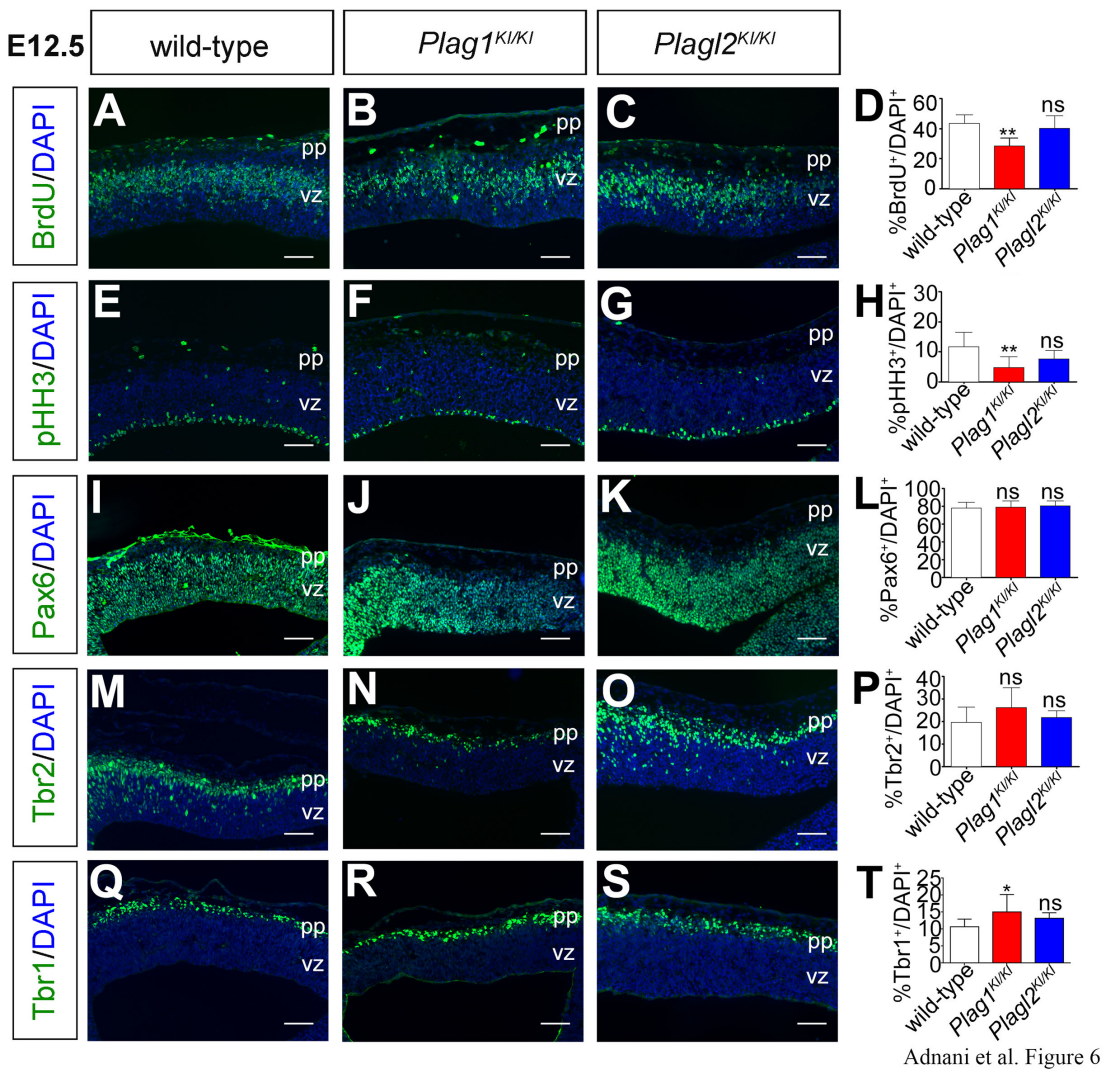
***Plag1* is required to regulate proliferation in the early embryonic telencephalon.** (A–T) Analysis of the expression of BrdU (A–C), pHH3 (E–G), Pax6 (I–K), Tbr2 (M–O) and Tbr1 (Q–S) in E12.5 wild-type (A,E,I,M,Q), *Plag1*^KI/KI^ (B,F,J,N,R) and *Plagl2*^KI/KI^ (C,G,K,O,S) cortices. Quantification of the percentage of DAPI^+^ cells expressing BrdU (D), pHH3 (H), Pax6 (L), Tbr2 (P) and Tbr1 (T). Error bars are s.e.m. ns, not significant; *P<0.05, **P<0.01, and ***P<0.005. pp, preplate; vz, ventricular zone. Scale bars: 125 μm.

To further assess the functions of the *Plag* genes in the developing telencephalon, we next focused on dorsal pallial territories, which give rise to the neocortex. To first ask whether *Plag1* and *Plagl2* were required to regulate the proliferation of neocortical progenitors, we administered BrdU 30 min prior to sacrifice to label progenitor cells in S-phase of the cell cycle. In E12.5 wild-type (Fig. 6A), *Plag1*^KI/KI^ (Fig. 6B) and *Plagl2*^KI/KI^ (Fig. 6C) cortices, BrdU was detected in an abventricular band where S-phase progenitors accumulate due to interkinetic nuclear migration. Quantitation revealed that there was a 1.67-fold reduction in the percentage of S-phase progenitors in the *Plag1*^KI/KI^ cortex (*n*=3; *P*<0.01), whereas there was no change in the BrdU labeling index in the *Plagl2*^KI/KI^ cortex (Fig. 6D). Similarly, labeling G2/M-phase progenitors with phospho-histone H3 (pHH3), which marks mitotic cells near the apical surface in E12.5 wild-type (Fig. 6E), *Plag1*^KI/KI^ (Fig. 6F) and *Plagl2*^KI/KI^ (Fig. 6G) cortices, revealed a threefold reduction in pHH3^+^ cells in *Plag1* mutants (Fig. 6H).

Taken together, these data suggest that *Plag1*, and not *Plagl2*, is required to regulate the number of cycling progenitors in S- and G2/M-phases of the cell cycle. We next assessed changes in the expression of progenitor cell markers in E12.5 wild-type (Fig. 6I,M), *Plag1*^KI/KI^ (Fig. 6J,N) and *Plagl2*^KI/KI^ (Fig. 6K,O) cortices, using Pax6 to label RGCs (Estivill-Torrus et al., 2002) and the T-box transcription factor Tbr2 to label INPs (Arnold et al., 2008; Sessa et al., 2008). Pax6 labeled the same number of RGC progenitors in the VZ in all genotypes (Fig. 6I–L), and Tbr2 labeled the same number of INPs (Fig. 6M–P). The decrease in VZ proliferation in *Plag1* mutants was therefore not translated into an overall change in progenitor cell number, at least by E12.5.

Changes in the BrdU and pHH3-labelling indices in *Plag1* mutants might reflect alterations in the ratios between proliferative and differentiating populations rather than changes in proliferation rates. We therefore examined whether *Plag1* and/or *Plagl2* regulated the differentiation of neocortical progenitors by examining the expression of Tbr1, a marker of early-born, deep-layer neurons (Hevner et al., 2001). There was a small but significant increase in the number of early-born neurons generated in E12.5 *Plag1*^KI/KI^ mutants compared to wild type, whereas there were no significant changes in Tbr1 expression in the *Plagl2*^KI/KI^ cortices relative to wild type (Fig. 6Q–T). Notably, the differences in proliferation and differentiation observed in *Plag1*^KI/KI^ mutants did not translate into alterations in the total number of DAPI^+^ nuclei in the E12.5 neocortex (wild type: 1393±76.4; *Plag1*^KI/KI^: 1188±68.8; *Plagl2*^KI/KI^: 1427±80.0; *n*=3 for each genotype).

Taken together we can conclude that *Plag1* is required to maintain the balanced choice between proliferation and differentiation in the E12.5 neocortex, suggesting that *Plagl2*, which is not mutated (or altered in expression) in *Plag1*^KI/KI^ embryos, is not sufficient to rescue this phenotype. In contrast, *Plagl2* is not required for the proliferation or differentiation of neocortical progenitors, possibly because *Plag1* and altered *Zac1* expression levels compensate to some extent for *Plagl2* functions.

### *Plagl2* increases the BrdU labelling index within 24 h post-electroporation

**Fig. 7.**
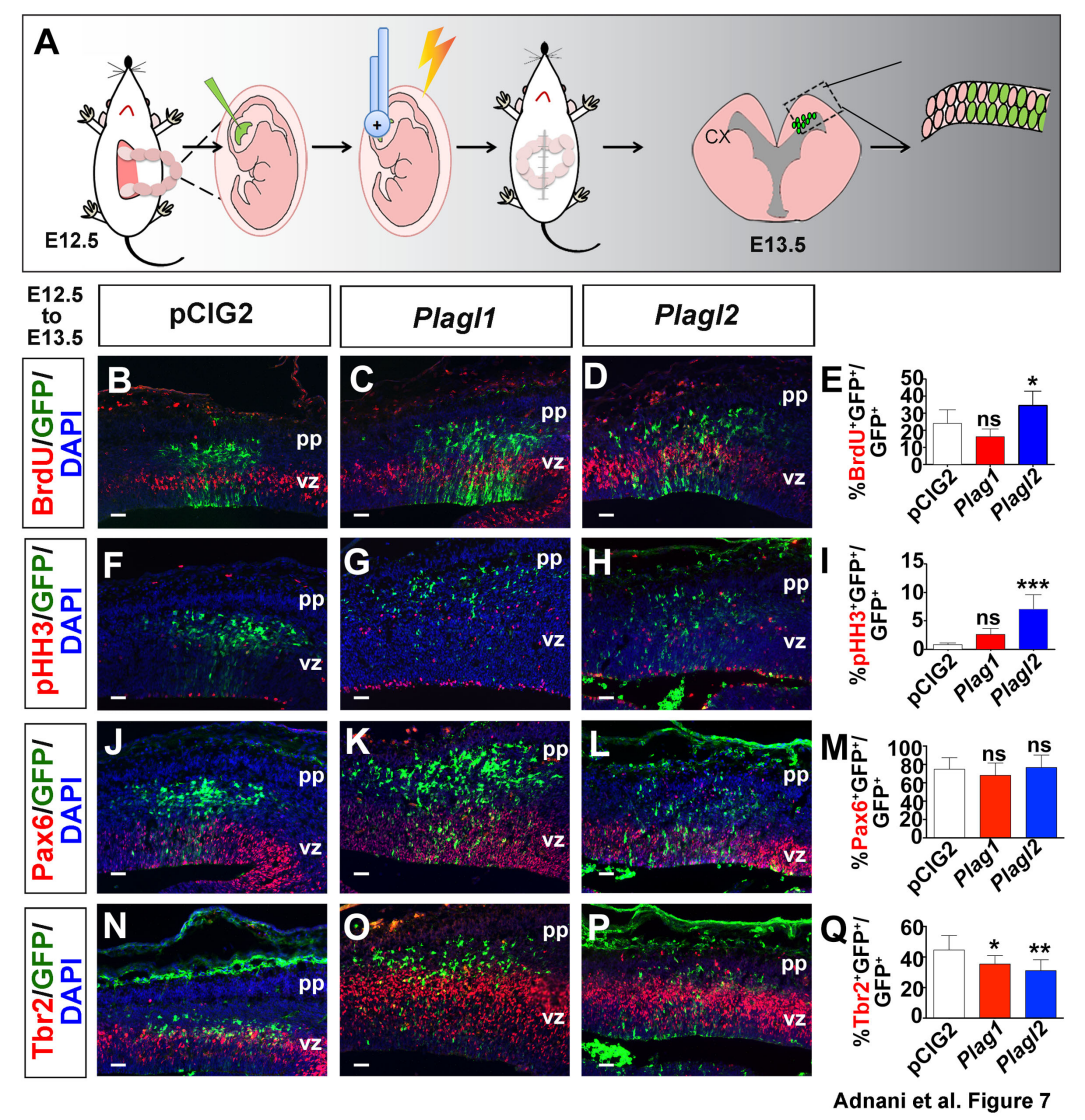
***Plagl2* is sufficient to alter the proliferation of neocortical progenitors within 24 h post-electroporation.** (A) Schematic representation of gain-of-function experiment using *in utero* electroporation. (B–Q) E12.5 to E13.5 electroporations of pCIG2 (B,F,J,N), *Plag1* (C,G,K,O) and *Plagl2* (D,H,L,P) analyzed for the expression of BrdU (B–D), pHH3 (F–H), Pax6 (J–L), Tbr2 (N–P). Quantitation of the ratios of GFP^+^ cells that are BrdU^+^ (E), pHH3^+^ (I), Pax6^+^ (M) and Tbr2^+^ (Q). Error bars are s.e.m. ns, not significant; *P<0.05, **P<0.01, and ***P<0.005. pp, preplate; vz, ventricular zone. Scale bars: 125 μm.

*Plag1* and *Plagl2* are both proto-oncogenes, promoting cell proliferation in a malignant context (Abdollahi, 2007; Van Dyck et al., 2007b). We therefore asked whether the overexpression of these factors in non-transformed, embryonic neural progenitors could similarly promote proliferation, and/or alter differentiation. For this purpose, we used *in utero* electroporation to introduce *Plag1* and/or *Plagl2* expression constructs containing an IRES-GFP cassette into dorsal telencephalic progenitors at E12.5, or an empty vector control expressing GFP only. Embryos were harvested 24 h post-electroporation and transfected cells were identified using GFP epifluorescence (Fig. 7A).

We first asked whether *Plag1* and/or *Plagl2* could promote ectopic proliferation by assessing the incorporation of BrdU administered 30 min before embryo collection. Relative to control E12.5→E13.5 electroporations, *Plagl2* (and not *Plag1*) was sufficient to increase the number of cells taking up BrdU (i.e. BrdU^+^GFP^+^/GFP^+^ cells; *n*=3; *P*<0.05; Fig. 7B–E). Similarly, *Plagl2* misexpression increased the number of cortical progenitors expressing pHH3, a G2/M-phase marker (*n*=3; *P*<0.001; Fig. 7F–I), whereas *Plag1* did not alter the pHH3^+^GFP^+^/GFP^+^ ratio. However, this increase in proliferation did not translate into an increase in RGC progenitor cells, as the ratio of Pax6^+^GFP^+^/GFP^+^ cells was not altered by either *Plag1* or *Plagl2* (*n*=3 each; Fig. 7J–M). Moreover, there was a decrease, rather than increase, in the number of Tbr2^+^GFP^+^/GFP^+^ INPs generated after the misexpression of both *Plag1* (*n*=3; *P*<0.05) and *Plagl2* (*n*=3; *P*<0.01) (Fig. 7N–Q).

In summary, *Plagl2* but not *Plag1* is sufficient to induce the proliferation of neocortical progenitors, at least within a short 24 h time window.

### *Plag1* and *Plagl2* inhibit neuronal differentiation 72 h post-electroporation, and *Plag1* has a delayed effect on increasing the BrdU labelling

**Fig. 8.**
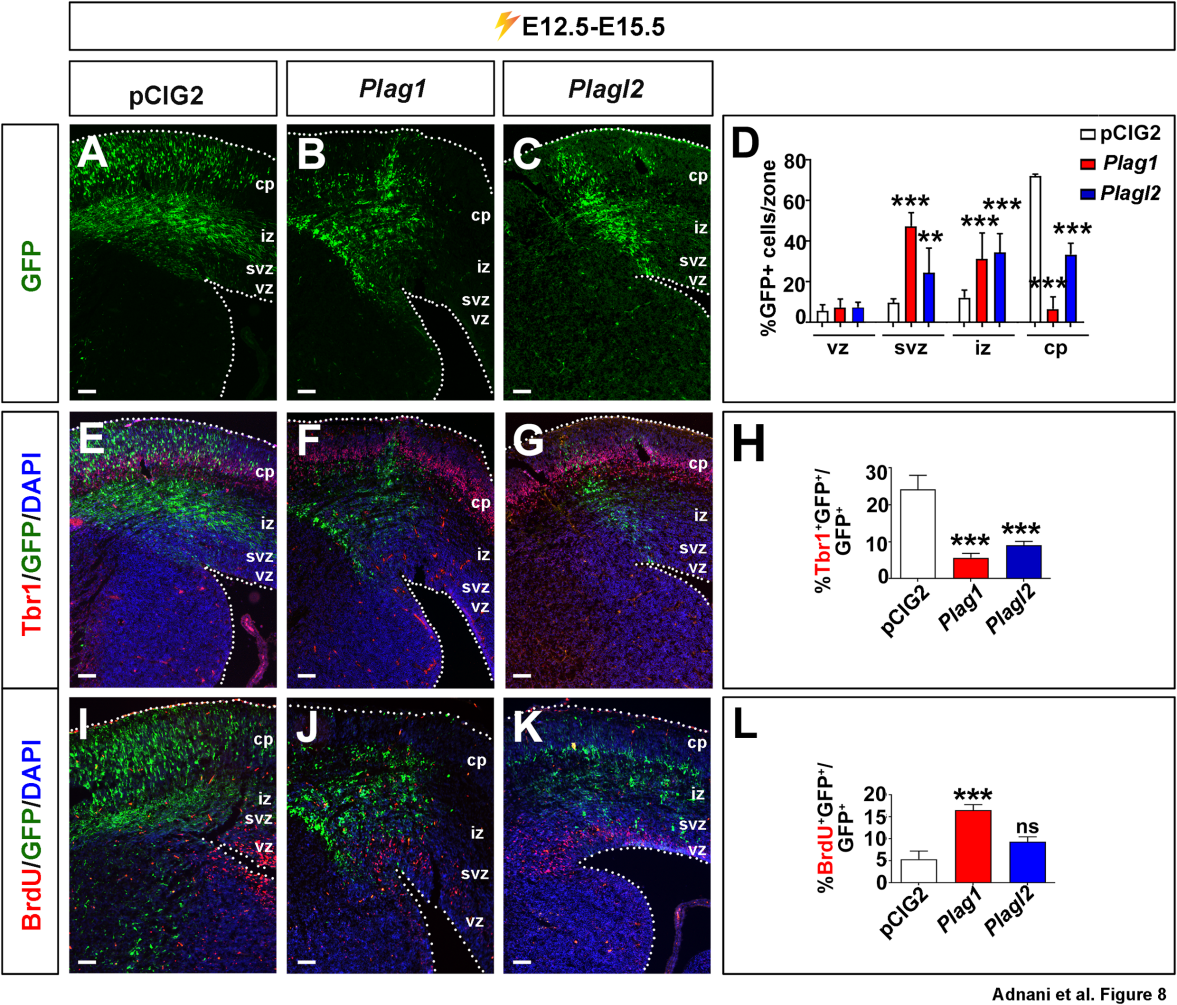
***Plag1* and *Plagl2* are sufficient to induce migration defects, and alter proliferation and differentiation when misexpressed in the neocortex 72 h post electroporation.** (A–L) E12.5 to E15.5 electroporations of pCIG2 (A,E,I), *Plag1* (B,F,J) and *Plagl2* (C,G,K) analyzed for the expression of GFP in different zones (A–D), and the co-expression of GFP with Tbr1 (E–H) and BrdU (I–L). Quantitation of the ratios of GFP^+^ cells that are in each zone (D), Tbr1^+^ (H) and BrdU^+^ (L). Error bars are s.e.m. ns, not significant; *P<0.05, **P<0.01, and ***P<0.005. cp, cortical plate; iz, intermediate zone; svz, subventricular zone; vz, ventricular zone. Scale bars: 125 μm.

To assess the effects of *Plag1* and *Plagl2* on neuronal differentiation and migration, we introduced *Plag1* and *Plagl2* expression vectors (and pCIG2 control) into E12.5 neocortical progenitors via *in utero* electroporation, but this time we assessed electroporated brains at E15.5, 72 h post-electroporation. The positions of the GFP^+^ electroporated cells were compared by counting labeled cells in the VZ, SVZ, intermediate zone (IZ) and cortical plate (CP). In control electroporations, most GFP^+^ labeled cells had already reached the CP 72 h post-electroporation (Fig. 8A,D). In contrast, E12.5→E15.5 electroporations of *Plag1* (Fig. 8B) and *Plagl2* (Fig. 8C) differed, in that more GFP^+^ electroporated cells were concentrated in the SVZ (*n*=3; *P*<0.001 for *Plag1* and *P*<0.01 for *Plagl2*; Fig. 8D) and IZ (*n*=3; *P*<0.001 for *Plag1* and *Plagl2*; Fig. 8D), and fewer GFP^+^ cells reached the CP (*n*=3; *P*<0.001 for *Plag1* and *Plagl2*; Fig. 8D). Overexpression of either *Plag1* or *Plagl2* thus strongly perturbs cellular migration, either because overexpressing cells fail to undergo neurogenesis, and/or because these genes impede neuronal migration.

To assess the effects of *Plag1* and *Plagl2* on neurogenesis in the E12.5 neocortex, at 72 h post-electroporation (at E15.5) we examined the expression of Tbr1 (Fig. 8E–G), which is expressed at high levels in deep-layer neocortical neurons (Englund et al., 2005). In E12.5→E15.5 electroporations the number of *Plag1-* and *Plagl2*-transfected cells that expressed Tbr1 was reduced compared to pCIG2 transfections (*n*=3; *P*<0.001 for both genes; Fig. 8E–H). These data suggested that *Plag1* and *Plagl2* do indeed block neuronal differentiation in the neocortex, and the inability of these cells to differentiate likely results in their migratory defects.

The inability of *Plagl2-*overexpressing progenitors to differentiate was consistent with the increased mitotic activity of these cells 24 h post-electroporation, whereas *Plag1* misexpression did not have the same effect after 24 h (Fig. 7E,I). To test whether *Plag1* and *Plagl2* influenced the proliferative capacity of E12.5 cortical progenitor cells 72 h post-electroporation, we performed a 30 min pulse-label with BrdU (Fig. 8I–L). In E12.5→E15.5 electroporations there were more GFP^+^BrdU^+^ S-phase progenitors in *Plag1* electroporations compared to pCIG2 control and *Plagl2* transfections (*n*=3; *P*<0.001; Fig. 8I–L). *Plag1*-misexpressing cells thus proliferate more and differentiate less, even though they take longer to initiate BrdU uptake compared to *Plagl2*-overexpressing cells.

## DISCUSSION

In this study we investigated the functions of the proto-oncogenes *Plag1* and *Plagl2* in the developing telencephalon. Our goal was to generate double mutants to assess genetic redundancy, but no live *Plag1;Plagl2* double-mutant embryos were collected at E12.5, indicating that these genes can compensate for one another with respect to overall embryonic growth and survival and that at least one gene copy of either *Plag1* or *Plagl2* is required. Furthermore, we found that the *Plagl2* genotype was early-embryonic lethal on a CD1 background, with a reduced number of single mutant embryos obtained at E12.5, restricting our analyses of telencephalic development to this early embryonic stage. Our analysis of E12.5 mutants revealed differences in the functions of *Plag1* and *Plagl2,* including an unexpected and striking patterning defect in the *Plagl2* mutant telencephalon, indicating that this gene is required to set the ventral boundary of dorsal gene expression. In contrast, *Plag1* is necessary to regulate the balance between proliferation and neuronal differentiation in the developing neocortex.

The *Plagl2* mutant patterning defect was somewhat unexpected as we observed an expansion of the ventral pallium without a corresponding decrease in the size of the dLGE, a territory immediately adjacent to the ventral pallium. This phenotype contrasts to defects observed upon the mutation of other patterning genes, including *Pax6* and *Gsx2*, which result in compensatory changes in the sizes of these two domains; in *Pax6* mutants, the dLGE expands at the expense of the ventral pallium, whereas the opposite phenotype is observed in *Gsx2* mutants (Yun et al., 2001). There is also a repression of a ventral pallial identity upon the deletion of *Nr2e1*, also called *Tlx* (*tailless*), resulting in the loss of *Sfrp1* and *Dbx1* expression (Stenman et al., 2003a,b). Complex genetic interactions thus regulate the establishment of these two important telencephalic domains, and *Plagl2* is an important determinant of ventral pallial domain size. Of note, while we did not observe a reduction in the number of Tbr1^+^ neurons in the presumptive piriform cortex in *Plagl2* mutants, which is derived from the ventral pallium, it may be that analyses at later stages could reveal defects. We were, however, precluded from these later studies by the early embryonic lethality of the *Plagl2* mutation.

While we did not observe patterning defects in *Plag1* mutants, we did observe that this gene is essential for neocortical progenitor proliferation and to block neuronal differentiation, consistent with its known role as a growth regulator. Interestingly, in gain-of-function studies, *Plag1* promotes BrdU uptake and reduces neurogenesis, but the effects on BrdU incorporation were delayed and only revealed 72 h post-electroporation. In contrast, *Plagl2* very rapidly induced BrdU uptake in E12.5 neocortical progenitors, within 24 h post-electroporation. While BrdU incorporation is a measure of all progenitors that are in S-phase of the cell cycle, it cannot be used to distinguish between those progenitors that are about to differentiate versus those that will continue to proliferate. However, taken together with the effects on differentiation, our data highly supports the idea that *Plag1* and *Plagl2* overexpression tips the balance towards more proliferative cell divisions in the early embryonic neocortex.

In summary, despite their related structures and roles as proto-oncogenes, there are key differences in how *Plag1* and *Plagl2* function *in vivo*. We discuss the potential reasons for these differences below, and compare *Plag1* and *Plagl2* functions to *Plagl1* (also known as *Zac1*), the third member of this gene family.

All three of the Plag proteins transactivate some common transcriptional targets, including several imprinted genes (e.g. *Dlk1*, *Igf2*) (Declercq et al., 2008; Van Dyck et al., 2008; Varrault et al., 2006; Voz et al., 2004; Zatkova et al., 2004), despite Plag1 and Plagl2 recognizing a distinct binding site (GRGGCN_6-8_G_3_) (Hensen et al., 2002; Kas et al., 1998; Voz et al., 2000) compared to Zac1 (G_4_C_4_, G_4_N_6_G_4_ or GC_2_GC_2_G) (Hoffmann et al., 2003; Varrault et al., 1998; Yuasa et al., 2010). Given the similar transcriptional targets, it is surprising that *Plag1* and *Plagl2* act as proto-oncogenes, while *Zac1* is a tumor suppressor gene. Moreover, *Zac1* misexpression was previously shown to reduce proliferation in the neocortex (Adnani et al., 2015; Rraklli et al., 2016), whereas in this study we showed that both *Plag1* and *Plagl2* were sufficient to promote BrdU uptake and block neurogenesis.

One possible reason for these functional differences despite overlapping transcriptional targets is that Zac1 can function as a transcriptional activator or repressor (Hoffmann et al., 2003; Varrault et al., 1998; Yuasa et al., 2010). When Zac1 binds as a monomer, it transactivates G_4_C_4_ and GC_2_GC_2_G sites and represses transcription from G_4_N_6_G_4_ sites, while dimer binding to G_4_N_6_G_4_ leads to transactivation (Hoffmann et al., 2003; Yuasa et al., 2010). Zac1 transcriptional activity is also modulated by interactions with nuclear importers (Huang et al., 2007), other transcription factors (Yuasa et al., 2010), and histone acetyltransferases (HAT) (Hoffmann et al., 2006). Zac1 can also act in a non-DNA binding-dependent manner, functioning as a co-activator or co-repressor of other transcriptional regulators [e.g. p53, nuclear receptors; Hoffmann et al. (2003); Huang et al. (2001); Huang and Stallcup (2000); Kas et al. (1998); Liu et al. (2008); Rozenfeld-Granot et al. (2002); Yuasa et al. (2010)].

In addition to sharing some common targets, the three transcription factors must also transactivate distinct genes in the developing telencephalon. Some studies have begun to identify transcriptional targets for *Plag1* (Voz et al., 2004), *Plagl2* (Zheng et al., 2010) and *Zac1* (Rraklli et al., 2016; Varrault et al., 2017, 2006), but which of these genes are important in telencephalic development will require further study. *Zac1* misexpression was shown to upregulate the expression of several imprinted genes, consistent with the finding that *Zac1* is part of an imprinted gene network (Varrault et al., 2006). *Zac1* also induced the expression of negative regulators of the cell cycle, such as the cyclin dependent kinase inhibitors p57 (which is imprinted) and p27, consistent with the ability of *Zac1* to promote cell cycle exit (Rraklli et al., 2016). Finally, *Zac1* misexpression also induced the ectopic expression of several genes not normally expressed in neural lineages, as well as genes associated with pluripotency, suggesting that one function of *Zac1* is to promote a pluripotent state (Rraklli et al., 2016). It will be of interest in the future to see whether *Plag1* and *Plagl2* also have a similar role in maintaining pluripotency.

We found that *Plag1* is required to regulate cell proliferation in the neocortex, which is of particular interest as several genome wide association studies (GWAS) have indicated that a SNP in *Plag1* is in one of 27 loci that correlate with height in humans (Yuasa et al., 2010), and additional studies have found correlations with stature or size in various livestock species, including cattle, pigs and horses (Juma et al., 2016). While these are association studies, the underlying assumption is that *Plag1* is an important regulator of growth. Interestingly, in our gain-of-function studies, *Plag1* was not sufficient to promote proliferation in neocortical progenitors, possibly because it acts in concert with other factors to carry out its growth regulatory role. Igf2 is a downstream transcriptional target of *Plag1* that has been implicated in growth control (Voz et al., 2000), and it may be that *Plag1* is not sufficient on its own to turn on this transcriptional target in the embryonic neocortex. Another possibility is that Plag1 is sumoylated in the embryonic neocortex, as this post-translational modification has been shown to repress the ability of Plag1 to transactivate downstream targets (Van Dyck et al., 2004).

In our study we found that *Plagl2* is sufficient but not necessary to promote the proliferation of neocortical progenitor cells. This data is consistent with a previous report showing that misexpression of *Plagl2* in *p53*^−/−^ adult neural stem cells promotes a proliferative phenotype when these cells are cultured *in vitro*, with the enriched expression of G1/S cell cycle checkpoint genes (Zheng et al., 2010). Moreover, we found that *Plagl2* reduces neuronal differentiation in the embryonic telencephalon, similar to how it functions in glioma cells (Zheng et al., 2010). These results suggest that *Plagl2* functions are context-dependent. Indeed, *Plagl2* is not oncogenic in all contexts [e.g. pro-apoptotic in response to hypoxia and other cellular stresses Furukawa et al. (2001); Guo et al. (2007); Mizutani et al. (2002); Yang et al. (2009)]. One reason why *Plagl2* functions may change in different contexts is that its transcriptional activity is also regulated by post-translational modifications, including sumoylation and acetylation (Guo et al., 2008; Ning et al., 2008; Van Dyck et al., 2004; Zheng and Yang, 2005). Under the right conditions, *Plagl2* may promote neocortical progenitor proliferation possibly via its capacity to initiate the transcription of Wnt pathway genes (Zheng et al., 2010), a key proliferative signal in the neocortex (Zheng et al., 2010).

In summary, our study reveals that *Plag1* and *Plagl2* are not only important regulators of tumorigenesis, but also play important redundant as well as complementary roles in normal CNS development and embryonic survival.

## MATERIALS AND METHODS

### Animals

The use of animals was vetted and approved by the University of Calgary and then the Sunnybrook Research Institute Animal Care Committees in agreement with the Guidelines of the Canadian Council of Animal Care (CCAC). The generation of *Plag1^lacZ^*^KI^ (Hensen et al., 2004) and *Plagl2^lacZ^*^KI^ (Van Dyck et al., 2007a) mice was previously reported and we maintained these mutant alleles on a CD1 background. For *Plag1* genotyping we used the following cycles: 95°C 4 min, 40× (95°C 1 min, 55°C 1 min, 72°C 1.5 min), 72°C 10 min. *Plag1* genotyping primers for the wild-type allele were: *Plag1* WT forward primer: 5′-CGGAAAGACCATCTGAAGAATCAC-3′. *Plag1* WT reverse primer: 5′-CGTTCGCAGTGCTCACATTG-3′. *Plag1* genotyping primers for the mutant allele were: *Plag1* mutant forward primer: 5′-CGGAAAGACCATCTGAAGAATCAC-3′. *Plag1* mutant reverse primer: 5′-AATGTGAGCGAGTAACAACCCG-3′. For *Plagl2* genotyping we used the following cycles: mutant: 95°C 4 min, 35× (95°C 1 min, 62°C 1 min, 72°C 1.5 min), 72°C 10 min. Wild type: 95°C 4 min, 40× (95°C 1 min, 59°C 1 min, 72°C 1.5 min), 72°C 10 min. *Plagl2* genotyping primers for the wild-type allele were: *Plagl2*WT forward primer: 5′-TGTATGGTGCCCACATCCCTAC-3′. *Plagl2*WT reverse primer: 5′-GGAAAAGTCCACATTAGCAGCG-3′. *Plagl2* genotyping primers for the mutant allele were: *Plagl2* MUT forward primer: 5′-CAGTTCAACATCAGCCGCTACAG-3′. *Plagl2* MUT reverse primer: 5′-GGTGGACAGTGGACATTTATCAAGG-3′.

### Tissue processing and cryostat sectioning

Embryos were dissected at E12.5 for all loss-of-function studies and at E13.5 for gain-of-function studies. Embryos were fixed overnight at 4°C in 4% paraformaldehyde (PFA) diluted in phosphate buffered saline (PBS) at pH 7.5. To remove the fixative, embryos were washed three times for 10 min in PBS, and then transferred to 20% sucrose/1× PBS overnight. For cyrosectioning, the brains were embedded in optimal cutting temperature (OCT) compound and stored at −80°C. Blocks were then sectioned at 10 µm on a cryostat for immunostaining and RNA *in situ* hybridization.

### Immunohistochemistry

For immunostaining, sections were blocked in 10% Horse Serum, 0.1% Triton-X100 in PBS (PBT) at pH 7.5 for 1 h. Primary antibodies were then diluted in blocking solution and the sections were incubated overnight at 4°C. Primary antibodies included rabbit anti-Tbr1 (1:800, Chemicon; Etobicoke, ON, Canada), rabbit anti-GFP (1:500, Chemicon, Temecula, CA, USA), goat-anti-GFP (1:1000, Abcam) rabbit anti-Pax6 (1:500, Convance), goat anti-Gsx2 (1:500, Millipore), rabbit anti-Tbr2 (1:500, Abcam), rabbit anti-phospho-histone H3 (pHH3; 1:500; Millipore Biotechnology) and rat anti-BrdU (1:20, Serotec). After incubating in primary antibody, the slides were washed three times in PBT and then incubated for 1 h at room temperature in secondary antibodies. Secondary antibodies were conjugated to Alexa568 (1:500; Molecular Probes) or Alexa488 (1:500; Molecular Probes). After incubation with secondary antibodies, the slides were washed three times in PBS and then stained with DAPI (1/10,000 for 5 min) and washed an additional three times. Slides were mounted in Aqua-polymount for imaging.

### BrdU labeling

100 µg/g body weight BrdU (Sigma-Aldrich) was injected intraperitoneally 30 min before the mice were euthanized. Prior to immunolabeling, sections were immersed in 2 N HCl for 15 min at 37°C (Britz et al., 2006). Immunolabeling was then performed as described.

### RNA *in situ* hybridization

RNA *in situ* hybridization was performed on 10 mm cryosections using a previously described protocol (Touahri et al., 2015). Riboprobes were described in the following publications; *Plag1* and *Plagl2* (Alam et al., 2005), *Dlx1* (Anderson et al., 1997), *Ascl1* (Cau et al., 1997), *Neurog2* (Gradwohl et al., 1996), *Etv1* (Di Meglio et al., 2008), *Sp8* (Waclaw et al., 2006) and *Dbx1* (Dixit et al., 2014).

### X-Gal staining

Slides were washed with DEPC phosphate buffered saline (PBS) pH7.5 for 5 min, three times. Sections were fixed in fixing solution (0.2% glutaraldehyde, 2% formaldehyde, 5 mM EGTA pH7.3, 2 mM magnesium chloride and 0.1 M sodium phosphate pH7.3 in PBS) for 15 min at room temperature. The slides were then washed with washing solution (0.02% NP40, 2 mM MgCl_2_ in PBS) for 10 min, three times. The slides were immersed in prewarmed staining solution [20 mg/ml X-gal-Sigma-Aldrich B4252 dissolved in DMSO, 5 mM K_3_Fe(CN)_6_, 5 mM K_4_Fe(CN)_6_, 2 mM MgCl_2_, 0.02% NP40 in PBS] and incubated in a 37°C water bath for 4 h to overnight protected from light. The tissues were dehydrated in 95% and 100% EtOH, and Xylene at room temperature. After the slides were dry, ∼4 drops of permount was added per slide and mounted with a coverslip for imaging.

### *In utero* electroporation

We performed *in utero* electroporation as previously described (Dixit et al., 2011; Langevin et al., 2007). Briefly, we introduced 3 μg/μl of a pCIG2 control vector, which expresses GFP, or 3 μg/μl of pCIG2-*Plag1* or pCIG2-*Plagl2*, which express the gene of interest and GFP, into E12.5 telencephalic vesicles using borosilicate needles and a Femtojet microinjector. Using a BTX electroporator, we applied 7 pulses of 55 mV within a 7 s interval to the uterus with the paddles flanking the head of the embryo. The uterus was then put back and embryos underwent normal development until E13.5. pCIG2-*Plag1* was generated by PCR amplification of *Plag1* from IMAGE clone ID 6328180 using the following primers: *Plag1*L: AATCTAGAGATGGCCACTGTCATTCCTGG; *Plag1*R: AATCTAGAGGCTACACAAGCA CCTCGGGT. The amplified *Plag1* cDNA was cloned as a blunted *Xba*I fragment into the blunted *EcoR*I site of pCIG2. pCIG2-*Plagl2* was generated by PCR amplification of *Plagl2* from IMAGE clone ID 6405960 using the following primers: *Plagl2*L: AATCTAGACATGACCACATTTTT CACCAG; *Plagl2*R: AATCTAGACTGAGTTGGGGGACCTTCAT. The amplified *Plagl2* cDNA was cloned was cloned directionally as an *EcoR*I fragment into the *EcoR*I site of pCIG2.

### RT-qPCR

We microdissected the dorsal telencephalon from E12.5 embryos as described. RNA was extracted with TRIzol reagent following the instructions of the manufacturer (Thermo Fisher Scientific, Cat#15596026). cDNA was synthesized and RT-qPCR was performed using a RT2 primer assay kit (Qiagen, 330001) according to the manufacturer's instructions. The following RT^2^ qPCR primers were obtained from Qiagen: *Gapdh* (PPM02946E), *B2m* (PPM03562A), *Hrpt* (PPM03559F), *Plag1* (PPM30678A), *Plagl2* (PPM30603B) and *Zac1* (PPM03537F). qPCR was performed with cortices from three embryos of each genotype and with three technical replicates for each biological replicate. We used the delta-delta Ct method to calculate relative expression levels, using three housekeeping genes to normalize (*Gapdh*, *B2M*, *Hrpt*).

### Imaging, quantitation and statistics

We captured images using OpenLab5 software (Improvision) and a QImaging RETIGA EX digital camera for bright-field images and a Leica DMRXA2 optical microscope for fluorescence imaging. Images were processed in Photoshop CS6 (64 bit; Adobe Systems) and quantification was performed from these images. For quantification we used a minimum of three embryos, and three sections per embryo. Calculation of statistical significance involved a one-way ANOVA with a Tukey correction for multiple comparisons. Graphs and statistical values were generated using GraphPad Prism software.

## References

[BIO038661C1] Abdollahi A. (2007). LOT1 (ZAC1/PLAGL1) and its family members: mechanisms and functions. *J. Cell. Physiol.* 210, 16-25. 10.1002/jcp.20835 17063461

[BIO038661C2] Abdollahi A., Godwin A. K., Miller P. D., Getts L. A., Schultz D. C., Taguchi T., Testa J. R. and Hamilton T. C. (1997a). Identification of a gene containing zinc-finger motifs based on lost expression in malignantly transformed rat ovarian surface epithelial cells. *Cancer Res.* 57, 2029-2034. 9158001

[BIO038661C3] Abdollahi A., Roberts D., Godwin A. K., Schultz D. C., Sonoda G., Testa J. R. and Hamilton T. C. (1997b). Identification of a zinc-finger gene at 6q25: a chromosomal region implicated in development of many solid tumors. *Oncogene* 14, 1973-1979. 10.1038/sj.onc.1201034 9150364

[BIO038661C4] Adnani L., Langevin L. M., Gautier E., Dixit R., Parsons K., Li S., Kaushik G., Wilkinson G., Wilson R., Childs S. et al. (2015). Zac1 regulates the differentiation and migration of neocortical neurons via Pac1. *J. Neurosci.* 35, 13430-13447. 10.1523/JNEUROSCI.0777-15.2015 26424889 PMC6605482

[BIO038661C5] Adnani L., Han S., Li S., Mattar P. and Schuurmans C. (2018). Mechanisms of cortical differentiation. *Int. Rev. Cell Mol. Biol.* 336, 223-320. 10.1016/bs.ircmb.2017.07.005 29413892

[BIO038661C6] Alam S., Zinyk D., Ma L. and Schuurmans C. (2005). Members of the Plag gene family are expressed in complementary and overlapping regions in the developing murine nervous system. *Dev. Dyn.* 234, 772-782. 10.1002/dvdy.20577 16193498

[BIO038661C7] Anderson S. A., Qiu M., Bulfone A., Eisenstat D. D., Meneses J., Pedersen R. and Rubenstein J. L. R. (1997). Mutations of the homeobox genes Dlx-1 and Dlx-2 disrupt the striatal subventricular zone and differentiation of late born striatal neurons. *Neuron* 19, 27-37. 10.1016/S0896-6273(00)80345-1 9247261

[BIO038661C8] Anderson A. A., Helmering J., Juan T., Li C.-M., McCormick J., Graham M., Baker D. M., Damore M. A., Véniant M. M. and Lloyd D. J. (2009). Pancreatic islet expression profiling in diabetes-prone C57BLKS/J mice reveals transcriptional differences contributed by DBA loci, including Plagl1 and Nnt. *Pathogenetics* 2, 1 10.1186/1755-8417-2-1 19161594 PMC2642818

[BIO038661C9] Arnold S. J., Huang G.-J., Cheung A. F. P., Era T., Nishikawa S.-I., Bikoff E. K., Molnar Z., Robertson E. J. and Groszer M. (2008). The T-box transcription factor Eomes/Tbr2 regulates neurogenesis in the cortical subventricular zone. *Genes Dev.* 22, 2479-2484. 10.1101/gad.475408 18794345 PMC2546697

[BIO038661C10] Asp J., Persson F., Kost-Alimova M. and Stenman G. (2006). CHCHD7-PLAG1 and TCEA1-PLAG1 gene fusions resulting from cryptic, intrachromosomal 8q rearrangements in pleomorphic salivary gland adenomas. *Genes Chromosomes Cancer* 45, 820-828. 10.1002/gcc.20346 16736500

[BIO038661C11] Astrom A. K., Voz M. L., Kas K., Roijer E., Wedell B., Mandahl N., Van de Ven W., Mark J. and Stenman G. (1999). Conserved mechanism of PLAG1 activation in salivary gland tumors with and without chromosome 8q12 abnormalities: identification of SII as a new fusion partner gene. *Cancer Res.* 59, 918-923. 10029085

[BIO038661C12] Astrom A., D'Amore E. S., Sainati L., Panarello C., Morerio C., Mark J. and Stenman G. (2000). Evidence of involvement of the PLAG1 gene in lipoblastomas. *Int. J. Oncol.* 16, 1107-1110. 10811981 10.3892/ijo.16.6.1107

[BIO038661C13] Basyuk E., Coulon V., Le Digarcher A., Coisy-Quivy M., Moles J. P., Gandarillas A. and Journot L. (2005). The candidate tumor suppressor gene ZAC is involved in keratinocyte differentiation and its expression is lost in basal cell carcinomas. *Mol. Cancer Res.* 3, 483-492. 10.1158/1541-7786.MCR-05-0019 16179495

[BIO038661C14] Bielle F., Griveau A., Narboux-Nême N., Vigneau S., Sigrist M., Arber S., Wassef M. and Pierani A. (2005). Multiple origins of Cajal-Retzius cells at the borders of the developing pallium. *Nat. Neurosci.* 8, 1002-1012. 10.1038/nn1511 16041369

[BIO038661C15] Bilanges B., Varrault A., Mazumdar A., Pantaloni C., Hoffmann A., Bockaert J., Spengler D. and Journot L. (2001). Alternative splicing of the imprinted candidate tumor suppressor gene ZAC regulates its antiproliferative and DNA binding activities. *Oncogene* 20, 1246-1253. 10.1038/sj.onc.1204237 11313869

[BIO038661C16] Britz O., Mattar P., Nguyen L., Langevin L. M., Zimmer C., Alam S., Guillemot F. and Schuurmans C. (2006). A role for proneural genes in the maturation of cortical progenitor cells. *Cereb. Cortex* 16 Suppl. 1, i138-i151. 10.1093/cercor/bhj168 16766700

[BIO038661C17] Casarosa S., Fode C. and Guillemot F. (1999). Mash1 regulates neurogenesis in the ventral telencephalon. *Development* 126, 525-534. 9876181 10.1242/dev.126.3.525

[BIO038661C18] Cau E., Gradwohl G., Fode C. and Guillemot F. (1997). Mash1 activates a cascade of bHLH regulators in olfactory neuron progenitors. *Development* 124, 1611-1621. 9108377 10.1242/dev.124.8.1611

[BIO038661C19] Chappell S. A., Walsh T., Walker R. A. and Shaw J. A. (1997). Loss of heterozygosity at chromosome 6q in preinvasive and early invasive breast carcinomas. *Br. J. Cancer* 75, 1324-1329. 10.1038/bjc.1997.224 9155053 PMC2228245

[BIO038661C20] Chung S.-H., Marzban H., Aldinger K., Dixit R., Millen K., Schuurmans C. and Hawkes R. (2011). Zac1 plays a key role in the development of specific neuronal subsets in the mouse cerebellum. *Neural Dev.* 6, 25 10.1186/1749-8104-6-25 21592321 PMC3113315

[BIO038661C21] Ciani E., Frenquelli M. and Contestabile A. (2003). Developmental expression of the cell cycle and apoptosis controlling gene, Lot1, in the rat cerebellum and in cultures of cerebellar granule cells. *Brain Res. Dev. Brain Res.* 142, 193-202. 10.1016/S0165-3806(03)00092-0 12711370

[BIO038661C22] Colitti C. V., Rodabaugh K. J., Welch W. R., Berkowitz R. S. and Mok S. C. (1998). A novel 4 cM minimal deletion unit on chromosome 6q25.1-q25.2 associated with high grade invasive epithelial ovarian carcinomas. *Oncogene* 16, 555-559. 10.1038/sj.onc.1201523 9484846

[BIO038661C23] Cvetkovic D., Pisarcik D., Lee C., Hamilton T. C. and Abdollahi A. (2004). Altered expression and loss of heterozygosity of the LOT1 gene in ovarian cancer. *Gynecol. Oncol.* 95, 449-455. 10.1016/j.ygyno.2004.08.051 15581945

[BIO038661C24] Czubryt M. P., Lamoureux L., Ramjiawan A., Abrenica B., Jangamreddy J. and Swan K. (2010). Regulation of cardiomyocyte Glut4 expression by ZAC1. *J. Biol. Chem.* 285, 16942-16950. 10.1074/jbc.M109.097246 20363751 PMC2878075

[BIO038661C25] Debiec-Rychter M., Van Valckenborgh I., Van den Broeck C., Hagemeijer A., Van de Ven W. J. M., Kas K., Van Damme B. and Voz M. L. (2001). Histologic localization of PLAG1 (pleomorphic adenoma gene 1) in pleomorphic adenoma of the salivary gland: cytogenetic evidence of common origin of phenotypically diverse cells. *Lab. Invest.* 81, 1289-1297. 10.1038/labinvest.3780342 11555676

[BIO038661C26] Declercq J., Hensen K., Van De Ven W. J. and Chavez M. (2003). PLAG proteins: how they influence apoptosis and cell proliferation. *Ann. N. Y. Acad. Sci.* 1010, 264-265. 10.1196/annals.1299.045 15033731

[BIO038661C27] Declercq J., Van Dyck F., Braem C. V., Van Valckenborgh I. C., Voz M., Wassef M., Schoonjans L., Van Damme B., Fiette L. and Van de Ven W. J. M. (2005). Salivary gland tumors in transgenic mice with targeted PLAG1 proto-oncogene overexpression. *Cancer Res.* 65, 4544-4553. 10.1158/0008-5472.CAN-04-4041 15930271

[BIO038661C28] Declercq J., Van Dyck F., Van Damme B. and Van de Ven W. J. (2008). Upregulation of Igf and Wnt signalling associated genes in pleomorphic adenomas of the salivary glands in PLAG1 transgenic mice. *Int. J. Oncol.* 32, 1041-1047. 10.3892/ijo.32.5.1041 18425330

[BIO038661C29] Di Meglio T., Nguyen-Ba-Charvet K. T., Tessier-Lavigne M., Sotelo C. and Chedotal A. (2008). Molecular mechanisms controlling midline crossing by precerebellar neurons. *J. Neurosci.* 28, 6285-6294. 10.1523/JNEUROSCI.0078-08.2008 18562598 PMC6670887

[BIO038661C30] Dixit R., Lu F., Cantrup R., Gruenig N., Langevin L. M., Kurrasch D. M. and Schuurmans C. (2011). Efficient gene delivery into multiple CNS territories using in utero electroporation. *J. Vis. Exp.*, Jun 23;(52). pii e2957 10.3791/2957 PMC3197065 21730943

[BIO038661C31] Dixit R., Wilkinson G., Cancino G. I., Shaker T., Adnani L., Li S., Dennis D., Kurrasch D., Chan J. A., Olson E. C. et al. (2014). Neurog1 and Neurog2 control two waves of neuronal differentiation in the piriform cortex. *J. Neurosci.* 34, 539-553. 10.1523/JNEUROSCI.0614-13.2014 24403153 PMC6608148

[BIO038661C32] Englund C., Fink A., Lau C., Pham D., Daza R. A., Bulfone A., Kowalczyk T. and Hevner R. F. (2005). Pax6, Tbr2, and Tbr1 are expressed sequentially by radial glia, intermediate progenitor cells, and postmitotic neurons in developing neocortex. *J. Neurosci.* 25, 247-251. 10.1523/JNEUROSCI.2899-04.2005 15634788 PMC6725189

[BIO038661C33] Enlund F., Nordkvist A., Sahlin P., Mark J. and Stenman G. (2002). Expression of PLAG1 and HMGIC proteins and fusion transcripts in radiation-associated pleomorphic adenomas. *Int. J. Oncol.* 20, 713-716. 10.3892/ijo.20.4.713 11894114

[BIO038661C34] Estivill-Torrus G., Pearson H., van Heyningen V., Price D. J. and Rashbass P. (2002). Pax6 is required to regulate the cell cycle and the rate of progression from symmetrical to asymmetrical division in mammalian cortical progenitors. *Development* 129, 455-466. 11807037 10.1242/dev.129.2.455

[BIO038661C35] Fode C., Ma Q., Casarosa S., Ang S. L., Anderson D. J. and Guillemot F. (2000). A role for neural determination genes in specifying the dorsoventral identity of telencephalic neurons. *Genes Dev.* 14, 67-80. 10640277 PMC316337

[BIO038661C36] Furukawa T., Adachi Y., Fujisawa J.-I., Kambe T., Yamaguchi-Iwai Y., Sasaki R., Kuwahara J., Ikehara S., Tokunaga R. and Taketani S. (2001). Involvement of PLAGL2 in activation of iron deficient- and hypoxia-induced gene expression in mouse cell lines. *Oncogene* 20, 4718-4727. 10.1038/sj.onc.1204647 11498794

[BIO038661C37] Gisselsson D., Hibbard M. K., Dal Cin P., Sciot R., Hsi B.-L., Kozakewich H. P. and Fletcher J. A. (2001). PLAG1 alterations in lipoblastoma: involvement in varied mesenchymal cell types and evidence for alternative oncogenic mechanisms. *Am. J. Pathol.* 159, 955-962. 10.1016/S0002-9440(10)61771-3 11549588 PMC1850475

[BIO038661C38] Gradwohl G., Fode C. and Guillemot F. (1996). Restricted expression of a novel murine atonal-related bHLH protein in undifferentiated neural precursors. *Dev. Biol.* 180, 227-241. 10.1006/dbio.1996.0297 8948587

[BIO038661C39] Guo Y., Yang M.-C., Weissler J. C. and Yang Y.-S. (2007). PLAGL2 translocation and SP-C promoter activity--a cellular response of lung cells to hypoxia. *Biochem. Biophys. Res. Commun.* 360, 659-665. 10.1016/j.bbrc.2007.06.106 17618602 PMC2084061

[BIO038661C40] Guo Y., Yang M.-C. W., Weissler J. C. and Yang Y.-S. (2008). Modulation of PLAGL2 transactivation activity by Ubc9 co-activation not SUMOylation. *Biochem. Biophys. Res. Commun.* 374, 570-575. 10.1016/j.bbrc.2008.07.064 18655774

[BIO038661C41] Han S., Dennis D. J., Balakrishnan A., Dixit R., Britz O., Zinyk D., Touahri Y., Olender T., Brand M., Guillemot F. et al. (2018). A non-canonical role for the proneural gene Neurog1 as a negative regulator of neocortical neurogenesis. *Development* 145, dev157719 10.1242/dev.157719 30201687 PMC6198467

[BIO038661C42] Hensen K., Van Valckenborgh I. C., Kas K., Van de Ven W. J. and Voz M. L. (2002). The tumorigenic diversity of the three PLAG family members is associated with different DNA binding capacities. *Cancer Res.* 62, 1510-1517. 11888928

[BIO038661C43] Hensen K., Braem C., Declercq J., Van Dyck F., Dewerchin M., Fiette L., Denef C. and Van de Ven W. J. M. (2004). Targeted disruption of the murine Plag1 proto-oncogene causes growth retardation and reduced fertility. *Dev. Growth Differ.* 46, 459-470. 10.1111/j.1440-169x.2004.00762.x 15606491

[BIO038661C44] Hevner R. F., Shi L., Justice N., Hsueh Y.-P., Sheng M., Smiga S., Bulfone A., Goffinet A. M., Campagnoni A. T. and Rubenstein J. L. R. (2001). Tbr1 regulates differentiation of the preplate and layer 6. *Neuron* 29, 353-366. 10.1016/S0896-6273(01)00211-2 11239428

[BIO038661C45] Hibbard M. K., Kozakewich H. P., Dal Cin P., Sciot R., Tan X., Xiao S. and Fletcher J. A. (2000). PLAG1 fusion oncogenes in lipoblastoma. *Cancer Res.* 60, 4869-4872. 10987300

[BIO038661C46] Hoch R. V., Rubenstein J. L. R. and Pleasure S. (2009). Genes and signaling events that establish regional patterning of the mammalian forebrain. *Semin. Cell Dev. Biol.* 20, 378-386. 10.1016/j.semcdb.2009.02.005 19560042

[BIO038661C47] Hoffmann A., Ciani E., Boeckardt J., Holsboer F., Journot L. and Spengler D. (2003). Transcriptional activities of the zinc finger protein Zac are differentially controlled by DNA binding. *Mol. Cell. Biol.* 23, 988-1003. 10.1128/MCB.23.3.988-1003.2003 12529403 PMC140694

[BIO038661C48] Hoffmann A., Barz T. and Spengler D. (2006). Multitasking C2H2 zinc fingers link Zac DNA binding to coordinated regulation of p300-histone acetyltransferase activity. *Mol. Cell. Biol.* 26, 5544-5557. 10.1128/MCB.02270-05 16809786 PMC1592709

[BIO038661C49] Huang S.-M. and Stallcup M. R. (2000). Mouse Zac1, a transcriptional coactivator and repressor for nuclear receptors. *Mol. Cell. Biol.* 20, 1855-1867. 10.1128/MCB.20.5.1855-1867.2000 10669760 PMC85366

[BIO038661C50] Huang S.-M., Schönthal A. H. and Stallcup M. R. (2001). Enhancement of p53-dependent gene activation by the transcriptional coactivator Zac1. *Oncogene* 20, 2134-2143. 10.1038/sj.onc.1204298 11360197

[BIO038661C51] Huang S.-M., Huang S.-P., Wang S.-L. and Liu P.-Y. (2007). Importin alpha1 is involved in the nuclear localization of Zac1 and the induction of p21WAF1/CIP1 by Zac1. *Biochem. J.* 402, 359-366. 10.1042/BJ20061295 17109628 PMC1798434

[BIO038661C52] Juma A. R., Damdimopoulou P. E., Grommen S. V. H., Van de Ven W. J. M. and De Groef B. (2016). Emerging role of PLAG1 as a regulator of growth and reproduction. *J. Endocrinol.* 228, R45-R56. 10.1530/JOE-15-0449 26577933

[BIO038661C53] Kamikihara T., Arima T., Kato K., Matsuda T., Kato H., Douchi T., Nagata Y., Nakao M. and Wake N. (2005). Epigenetic silencing of the imprinted gene ZAC by DNA methylation is an early event in the progression of human ovarian cancer. *Int. J. Cancer* 115, 690-700. 10.1002/ijc.20971 15751035

[BIO038661C54] Kandasamy J., Smith A., Diaz S., Rose B. and O'Brien C. (2007). Heterogeneity of PLAG1 gene rearrangements in pleomorphic adenoma. *Cancer Genet. Cytogenet.* 177, 1-5. 10.1016/j.cancergencyto.2007.04.006 17693184

[BIO038661C55] Kas K., Voz M. L., Roijer E., Åström A.-K., Meyen E., Stenman G. and Van de Ven W. J. M. (1997). Promoter swapping between the genes for a novel zinc finger protein and beta-catenin in pleiomorphic adenomas with t(3;8)(p21;q12) translocations. *Nat. Genet.* 15, 170-174. 10.1038/ng0297-170 9020842

[BIO038661C56] Kas K., Voz M. L., Hensen K., Meyen E. and Van de Ven W. J. M. (1998). Transcriptional activation capacity of the novel PLAG family of zinc finger proteins. *J. Biol. Chem.* 273, 23026-23032. 10.1074/jbc.273.36.23026 9722527

[BIO038661C57] Koy S., Hauses M., Appelt H., Friedrich K., Schackert H. K. and Eckelt U. (2004). Loss of expression of ZAC/LOT1 in squamous cell carcinomas of head and neck. *Head Neck* 26, 338-344. 10.1002/hed.10386 15054737

[BIO038661C58] Landrette S. F., Kuo Y. H., Hensen K., Barjesteh van Waalwijk van Doorn-Khosrovani S., Perrat P. N., Van de Ven W. J., Delwel R. and Castilla L. H. (2005). Plag1 and Plagl2 are oncogenes that induce acute myeloid leukemia in cooperation with Cbfb-MYH11. *Blood* 105, 2900-2907. 10.1182/blood-2004-09-3630 15585652

[BIO038661C59] Langevin L. M., Mattar P., Scardigli R., Roussigné M., Logan C., Blader P. and Schuurmans C. (2007). Validating in utero electroporation for the rapid analysis of gene regulatory elements in the murine telencephalon. *Dev. Dyn.* 236, 1273-1286. 10.1002/dvdy.21126 17377980

[BIO038661C60] Lemeta S., Jarmalaite S., Pylkkänen L., Böhling T. and Husgafvel-Pursiainen K. (2007). Preferential loss of the nonimprinted allele for the ZAC1 tumor suppressor gene in human capillary hemangioblastoma. *J. Neuropathol. Exp. Neurol.* 66, 860-867. 10.1097/nen.0b013e318149ee64 17805016

[BIO038661C61] Liu P.-Y., Chan J. Y.-H., Lin H.-C., Wang S.-L., Liu S.-T., Ho C.-L., Chang L.-C. and Huang S.-M. (2008). Modulation of the cyclin-dependent kinase inhibitor p21(WAF1/Cip1) gene by Zac1 through the antagonistic regulators p53 and histone deacetylase 1 in HeLa Cells. *Mol. Cancer Res.* 6, 1204-1214. 10.1158/1541-7786.MCR-08-0123 18644983

[BIO038661C62] Ma L., Cantrup R., Varrault A., Colak D., Klenin N., Götz M., McFarlane S., Journot L. and Schuurmans C. (2007a). Zac1 functions through TGFbetaII to negatively regulate cell number in the developing retina. *Neural Dev.* 2, 11 10.1186/1749-8104-2-11 17559664 PMC1913510

[BIO038661C63] Ma L., Hocking J. C., Hehr C. L., Schuurmans C. and McFarlane S. (2007b). Zac1 promotes a Müller glial cell fate and interferes with retinal ganglion cell differentiation in Xenopus retina. *Dev. Dyn.* 236, 192-202. 10.1002/dvdy.21002 17072860

[BIO038661C64] Mizutani A., Furukawa T., Adachi Y., Ikehara S. and Taketani S. (2002). A zinc-finger protein, PLAGL2, induces the expression of a proapoptotic protein Nip3, leading to cellular apoptosis. *J. Biol. Chem.* 277, 15851-15858. 10.1074/jbc.M111431200 11832486

[BIO038661C65] Morerio C., Rapella A., Rosanda C., Tassano E., Gambini C., Romagnoli G. and Panarello C. (2005). PLAG1-HAS2 fusion in lipoblastoma with masked 8q intrachromosomal rearrangement. *Cancer Genet. Cytogenet.* 156, 183-184. 10.1016/j.cancergencyto.2004.04.017 15642402

[BIO038661C66] Ning J., Zheng G. and Yang Y.-C. (2008). Tip60 modulates PLAGL2-mediated transactivation by acetylation. *J. Cell. Biochem.* 103, 730-739. 10.1002/jcb.21444 17551969

[BIO038661C67] Pagotto U., Arzberger T., Ciani E., Lezoualc'h F., Pilon C., Journot L., Spengler D. and Stalla G. K. (1999). Inhibition of Zac1, a new gene differentially expressed in the anterior pituitary, increases cell proliferation. *Endocrinology* 140, 987-996. 10.1210/endo.140.2.6532 9927333

[BIO038661C68] Pagotto U., Arzberger T., Theodoropoulou M., Grubler Y., Pantaloni C., Saeger W., Losa M., Journot L., Stalla G. K. and Spengler D. (2000). The expression of the antiproliferative gene ZAC is lost or highly reduced in nonfunctioning pituitary adenomas. *Cancer Res.* 60, 6794-6799. 11156367

[BIO038661C69] Pallasch C. P., Patz M., Park Y. J., Hagist S., Eggle D., Claus R., Debey-Pascher S., Schulz A., Frenzel L. P., Claasen J. et al. (2009). miRNA deregulation by epigenetic silencing disrupts suppression of the oncogene PLAG1 in chronic lymphocytic leukemia. *Blood* 114, 3255-3264. 10.1182/blood-2009-06-229898 19692702 PMC2925729

[BIO038661C70] Poulin H. and Labelle Y. (2005). The PLAGL1 gene is down-regulated in human extraskeletal myxoid chondrosarcoma tumors. *Cancer Lett.* 227, 185-191. 10.1016/j.canlet.2004.12.007 16112421

[BIO038661C71] Rodríguez-Henche N., Jamen F., Leroy C., Bockaert J. and Brabet P. (2002). Transcription of the mouse PAC1 receptor gene: cell-specific expression and regulation by Zac1. *Biochim. Biophys. Acta* 1576, 157-162. 10.1016/S0167-4781(02)00303-2 12031496

[BIO038661C72] Röpke A., Kalinski T., Kluba U., von Falkenhausen U., Wieacker P. F. and Röpke M. (2007). PLAG1 activation in lipoblastoma coinciding with low-level amplification of a derivative chromosome 8 with a deletion del(8)(q13q21.2). *Cytogenet Genome Res.* 119, 33-38. 10.1159/000109616 18160779

[BIO038661C73] Rozenfeld-Granot G., Krishnamurthy J., Kannan K., Toren A., Amariglio N., Givol D. and Rechavi G. (2002). A positive feedback mechanism in the transcriptional activation of Apaf-1 by p53 and the coactivator Zac-1. *Oncogene* 21, 1469-1476. 10.1038/sj.onc.1205218 11896574

[BIO038661C74] Rraklli V., Södersten E., Nyman U., Hagey D. W. and Holmberg J. (2016). Elevated levels of ZAC1 disrupt neurogenesis and promote rapid in vivo reprogramming. *Stem Cell Res.* 16, 1-9. 10.1016/j.scr.2015.11.002 26610203

[BIO038661C75] Schuurmans C. and Guillemot F. (2002). Molecular mechanisms underlying cell fate specification in the developing telencephalon. *Curr. Opin. Neurobiol.* 12, 26-34. 10.1016/S0959-4388(02)00286-6 11861161

[BIO038661C76] Sessa A., Mao C.-A., Hadjantonakis A.-K., Klein W. H. and Broccoli V. (2008). Tbr2 directs conversion of radial glia into basal precursors and guides neuronal amplification by indirect neurogenesis in the developing neocortex. *Neuron* 60, 56-69. 10.1016/j.neuron.2008.09.028 18940588 PMC2887762

[BIO038661C77] Spengler D., Villalba M., Hoffmann A., Pantaloni C., Houssami S., Bockaert J. and Journot L. (1997). Regulation of apoptosis and cell cycle arrest by Zac1, a novel zinc finger protein expressed in the pituitary gland and the brain. *EMBO J.* 16, 2814-2825. 10.1093/emboj/16.10.2814 9184226 PMC1169890

[BIO038661C78] Stenman J., Yu R. T., Evans R. M. and Campbell K. (2003a). Tlx and Pax6 co-operate genetically to establish the pallio-subpallial boundary in the embryonic mouse telencephalon. *Development* 130, 1113-1122. 10.1242/dev.00328 12571103

[BIO038661C79] Stenman J. M., Wang B. and Campbell K. (2003b). Tlx controls proliferation and patterning of lateral telencephalic progenitor domains. *J. Neurosci.* 23, 10568-10576. 10.1523/JNEUROSCI.23-33-10568.2003 14627641 PMC6740920

[BIO038661C80] Theile M., Seitz S., Arnold W., Jandrig B., Frege R., Schlag P. M., Haensch W., Guski H., Winzer K. J., Barrett J. C. et al. (1996). A defined chromosome 6q fragment (at D6S310) harbors a putative tumor suppressor gene for breast cancer. *Oncogene* 13, 677-685. 8761288

[BIO038661C81] Theodoropoulou M., Zhang J., Laupheimer S., Paez-Pereda M., Erneux C., Florio T., Pagotto U. and Stalla G. K. (2006). Octreotide, a somatostatin analogue, mediates its antiproliferative action in pituitary tumor cells by altering phosphatidylinositol 3-kinase signaling and inducing Zac1 expression. *Cancer Res.* 66, 1576-1582. 10.1158/0008-5472.CAN-05-1189 16452215

[BIO038661C82] Theodoropoulou M., Tichomirowa M. A., Sievers C., Yassouridis A., Arzberger T., Hougrand O., Deprez M., Daly A. F., Petrossians P., Pagotto U. et al. (2009). Tumor ZAC1 expression is associated with the response to somatostatin analog therapy in patients with acromegaly. *Int. J. Cancer* 125, 2122-2126. 10.1002/ijc.24602 19637311

[BIO038661C83] Theodoropoulou M., Stalla G. K. and Spengler D. (2010). ZAC1 target genes and pituitary tumorigenesis. *Mol. Cell. Endocrinol.***326**, 60-65. 10.1016/j.mce.2010.01.033 20117169

[BIO038661C84] Touahri Y., Adnani L., Mattar P., Markham K., Klenin N. and Schuurmans C. (2015). Non-isotopic RNA in situ hybridization on embryonic sections. *Curr. Protoc. Neurosci.* 70, 1 22 21-1 22 25. 10.1002/0471142301.ns0122s70 25559002

[BIO038661C85] Valente T., Junyent F. and Auladell C. (2005). Zac1 is expressed in progenitor/stem cells of the neuroectoderm and mesoderm during embryogenesis: differential phenotype of the Zac1-expressing cells during development. *Dev. Dyn.* 233, 667-679. 10.1002/dvdy.20373 15844099

[BIO038661C86] Van Dyck F., Delvaux E. L. D., Van De Ven W. J. M. and Chavez M. V. (2004). Repression of the transactivating capacity of the oncoprotein PLAG1 by SUMOylation. *J. Biol. Chem.* 279, 36121-36131. 10.1074/jbc.M401753200 15208321

[BIO038661C87] Van Dyck F., Braem C. V., Chen Z., Declercq J., Deckers R., Kim B.-M., Ito S., Wu M. K., Cohen D. E., Dewerchin M. et al. (2007a). Loss of the PlagL2 transcription factor affects lacteal uptake of chylomicrons. *Cell Metab.* 6, 406-413. 10.1016/j.cmet.2007.09.010 17983586

[BIO038661C88] Van Dyck F., Declercq J., Braem C. V. and Van de Ven W. J. (2007b). PLAG1, the prototype of the PLAG gene family: versatility in tumour development (review). *Int. J. Oncol.* 30, 765-774. 10.3892/ijo.30.4.765 17332914

[BIO038661C89] Van Dyck F., Scroyen I., Declercq J., Sciot R., Kahn B., Lijnen R. and Van de Ven W. J. (2008). aP2-Cre-mediated expression activation of an oncogenic PLAG1 transgene results in cavernous angiomatosis in mice. *Int. J. Oncol.* 32, 33-40. 10.3892/ijo.32.1.33 18097540

[BIO038661C90] Varrault A., Ciani E., Apiou F., Bilanges B., Hoffmann A., Pantaloni C., Bockaert J., Spengler D. and Journot L. (1998). hZAC encodes a zinc finger protein with antiproliferative properties and maps to a chromosomal region frequently lost in cancer. *Proc. Natl. Acad. Sci. USA* 95, 8835-8840. 10.1073/pnas.95.15.8835 9671765 PMC21163

[BIO038661C91] Varrault A., Gueydan C., Delalbre A., Bellmann A., Houssami S., Aknin C., Severac D., Chotard L., Kahli M., Le Digarcher A. et al. (2006). Zac1 regulates an imprinted gene network critically involved in the control of embryonic growth. *Dev. Cell* 11, 711-722. 10.1016/j.devcel.2006.09.003 17084362

[BIO038661C92] Varrault A., Dantec C., Le Digarcher A., Chotard L., Bilanges B., Parrinello H., Dubois E., Rialle S., Severac D., Bouschet T. et al. (2017). Identification of Plagl1/Zac1 binding sites and target genes establishes its role in the regulation of extracellular matrix genes and the imprinted gene network. *Nucleic Acids Res.* 45, 10466-10480. 10.1093/nar/gkx672 28985358 PMC5737700

[BIO038661C93] Voz M. L., Åström A.-K., Kas K., Mark J., Stenman G. and Van de Ven W. J. M. (1998). The recurrent translocation t(5;8)(p13;q12) in pleomorphic adenomas results in upregulation of PLAG1 gene expression under control of the LIFR promoter. *Oncogene* 16, 1409-1416. 10.1038/sj.onc.1201660 9525740

[BIO038661C94] Voz M. L., Agten N. S., Van de Ven W. J. and Kas K. (2000). PLAG1, the main translocation target in pleomorphic adenoma of the salivary glands, is a positive regulator of IGF-II. *Cancer Res.* 60, 106-113. 10646861

[BIO038661C95] Voz M. L., Mathys J., Hensen K., Pendeville H., Van Valckenborgh I., Van Huffel C., Chavez M., Van Damme B., De Moor B., Moreau Y. et al. (2004). Microarray screening for target genes of the proto-oncogene PLAG1. *Oncogene* 23, 179-191. 10.1038/sj.onc.1207013 14712223

[BIO038661C96] Waclaw R. R., Allen Z. J. II, Bell S. M., Erdélyi F., Szabó G., Potter S. S. and Campbell K. (2006). The zinc finger transcription factor Sp8 regulates the generation and diversity of olfactory bulb interneurons. *Neuron* 49, 503-516. 10.1016/j.neuron.2006.01.018 16476661

[BIO038661C97] Wilkinson G., Dennis D. and Schuurmans C. (2013). Proneural genes in neocortical development. *Neuroscience* 253, 256-273. 10.1016/j.neuroscience.2013.08.029 23999125

[BIO038661C98] Yang Y.-S., Yang M.-C. W., Guo Y., Williams O. W. and Weissler J. C. (2009). PLAGL2 expression-induced lung epithelium damages at bronchiolar alveolar duct junction in emphysema: bNip3- and SP-C-associated cell death/injury activity. *Am. J. Physiol. Lung Cell. Mol. Physiol.* 297, L455-L466. 10.1152/ajplung.00144.2009 19574421 PMC2739772

[BIO038661C99] Yuasa S., Onizuka T., Shimoji K., Ohno Y., Kageyama T., Yoon S. H., Egashira T., Seki T., Hashimoto H., Nishiyama T. et al. (2010). Zac1 is an essential transcription factor for cardiac morphogenesis. *Circ. Res.* 106, 1083-1091. 10.1161/CIRCRESAHA.109.214130 20167925

[BIO038661C100] Yun K., Potter S. and Rubenstein J. L. (2001). Gsh2 and Pax6 play complementary roles in dorsoventral patterning of the mammalian telencephalon. *Development* 128, 193-205. 11124115 10.1242/dev.128.2.193

[BIO038661C101] Zatkova A., Rouillard J.-M., Hartmann W., Lamb B. J., Kuick R., Eckart M., von Schweinitz D., Koch A., Fonatsch C., Pietsch T. et al. (2004). Amplification and overexpression of the IGF2 regulator PLAG1 in hepatoblastoma. *Genes Chromosomes Cancer* 39, 126-137. 10.1002/gcc.10307 14695992

[BIO038661C102] Zhao X., Ren W., Yang W., Wang Y., Kong H., Wang L., Yan L., Xu G., Fei J., Fu J. et al. (2006). Wnt pathway is involved in pleomorphic adenomas induced by overexpression of PLAG1 in transgenic mice. *Int. J. Cancer* 118, 643-648. 10.1002/ijc.21400 16108035

[BIO038661C103] Zheng G. and Yang Y.-C. (2005). Sumoylation and acetylation play opposite roles in the transactivation of PLAG1 and PLAGL2. *J. Biol. Chem.* 280, 40773-40781. 10.1074/jbc.M504334200 16207715

[BIO038661C104] Zheng H., Ying H., Wiedemeyer R., Yan H., Quayle S. N., Ivanova E. V., Paik J.-H., Zhang H., Xiao Y., Perry S. R. et al. (2010). PLAGL2 regulates Wnt signaling to impede differentiation in neural stem cells and gliomas. *Cancer Cell* 17, 497-509. 10.1016/j.ccr.2010.03.020 20478531 PMC2900858

